# WOODIV v2, more occurrences, functional traits, and a time-calibrated phylogeny for Euro-Mediterranean trees

**DOI:** 10.1038/s41597-025-06050-0

**Published:** 2025-11-06

**Authors:** Manuel Cartereau, Geordie Biffoni, Alex Baumel, Gianluigi Bacchetta, Lenka Brousset, Giacomo Calvia, Gabriele Casazza, Angelo Costanzo, Maria Guerrina, Antonino Modaffari, Virgile Noble, Lorenzo Peruzzi, Daniel Pavon, Marco Porceddu, Vedran Šegota, Elysa Silva, Francesco Todaro, Nina Vuković, Agathe Leriche

**Affiliations:** 1 Aix Marseille Univ, Avignon Univ, CNRS, IRD, IMBE, Aix-en-Provence, France; 2 DISTAV, University of Genova, Corso Europa 26, 16132 Genova, Italy; 3 Sardinian Germplasm Bank (BG-SAR), Centre for Conservation of Biodiversity (CCB), Department of Life and Environmental Sciences, University of Cagliari, Viale Sant’Ignazio da Laconi 9-13, 09123 Cagliari, Italy; 4 Free University of Bozen-Bolzano, Faculty of Agricultural, Environmental and Food Sciences, Piazza Università, 5, 39100 Bozen-Bolzano, Italy; 5 Conservatoire Botanique National Méditerranéen, Hyères, France; 6 PLANTSEED Lab, Department of Biology, University of Pisa, Via Derna 1, 56126 Pisa, Italy; 7 Faculty of Science, Department of Biology, University of Zagreb, Horvatovac 102a, Zagreb, Croatia; 8 Department of Ecology, University of Alicante, Spain, Institute of multidisciplinary environmental studies “Ramón Margalef”, University of Alicante, Alicante, Spain; 9 Department of Biology, Faculty of Science, University of Zagreb, Rooseveltov Trg 6, 10000 Zagreb, Croatia

**Keywords:** Biodiversity, Macroecology, Community ecology, Biodiversity, Forest ecology

## Abstract

The WOODIV v1 database provided occurrences at 10 km spatial resolution, traits and phylogenetic data for all 210 species (and 35 subspecies, totalling 245 taxa) in the Euro-Mediterranean Basin. While this reliable and readily accessible database has been of crucial help to investigate macro-ecological and biodiversity conservation questions, important knowledge gaps remained. To fill these gaps, we present an updated and extended version of the WOODIV database. (i) Occurrence data were updated from sources already considered in v1, three additional sources and an intensive field work campaign in Italy where occurrence data were scarce in v1, allowing to highly increase spatial coverage and data completeness. (ii) Besides increasing taxonomic coverage of traits already considered in v1, the traits data set has been extended to 15 new functional traits encompassing morpho-anatomy, reproduction and phenology, using new traits measurements from the field and data compilation from botanical literature not available in other databases. (iii) Finally, a new time-calibrated phylogeny for all 210 tree species was built and made available.

## Background & Summary

Forests play a crucial part in sustaining life on earth, *e.g*., preventing soil erosion, acting as storehouses of carbon, providing food and fuel to human populations and hosting high levels of biodiversity^1,2^. Higher levels of ecosystem services characterize woodlands with a higher tree species diversity^3^. In a context of global warming and land-use intensification, it is therefore crucial to have reliable data on the composition, functioning and diversity of woodland ecosystems. This is particularly true for the Euro-Mediterranean region, recognized by global conservation assessments as a priority for the conservation of the world’s biodiversity. Indeed, the Mediterranean biome offers a unique diversity of habitats, particularly threatened^4^, but should experience drastic biodiversity changes by 2100 owing to its sensitivity to land use change (due to ongoing increased population) and climate change^5,6^.

Despite being essential for biogeography and conservation, data on tree species in the Euro-Mediterranean region were sparse, uneven, and hard to access: the first version of the WOODIV database^7^ (WOODIV v1) started to fill this gap and has been used to address macroecological-biogeographical and methodological questions^8–10^. WOODIV v1 provides 171,711 records (unique combination of species, grid cell and source of data) and 148,649 occurrences (unique combination of species and grid cell) for the Euro-Mediterranean tree species of the Médail *et al*.^11^ checklist, at 10 × 10 km resolution (i.e., INSPIRE 10 × 10 km equal-area grid, EPSG 3035), gathered from 23 different sources together with data from field work (Montenegro) and expert knowledge (Sicily, Sardinia, Northern Macedonia and Malta). Validation analyses showed a strong input of the WOODIV v1 database for Euro-Mediterranean trees distribution knowledge, but highlighted the existence of some gaps, especially in Italy, where most existing data were only available in grey literature that is neither structured nor accessible. Values for only 4 functional traits were provided in WOODIV v1, and not for all considered species: adult plant height (available for 201 species out of the 210 considered), seed mass (159 species), stem specific density (114 species), and specific leaf area (102 species). These four traits were chosen because they have been proposed to reflect the global spectrum of plant strategies^12,13^, but they do not fully capture other aspects of plant strategies such as reproduction and dispersion^14,15^. Finally, WOODIV v1 provides data for three DNA markers and an associated phylogram including the 204 species (among the 210 considered) for which at least one DNA sequence was available. Although this phylogenetic tree provides a reliable topology, divergence timing was not provided.

To overcome these deficiencies in WOODIV v1, we have created an extended and updated version of the database (WOODIV v2), still considering the 210 Euro-Mediterranean tree species identified by Médail *et al*.^11^; including the 44 cryptic tree species which are often neglected in existing forest databases, at 10 × 10 km spatial resolution. This uses the same basic structure as the previous version, with additional fields to accommodate new data. As a result of intensive field campaigns carried out as part of the INTEGRADIV project (Biodiversa + funding), as well as the updating of data sources and the addition of new ones, WOODIV v2 provides much better coverage of tree occurrences throughout the Euro-Mediterranean region, and particularly in Italy (including Sicily and Sardinia) and Spain. Availability of data for three functional traits considered in WOODIV v1 (seed mass, stem specific density and specific leaf area) also largely increases especially thanks to the field-sampling campaigns. We also considered 15 new functional traits related to phenology, reproduction and morpho-anatomy of trees, with values provided in WOODIV v2 gathered from multiple sources. Finally, WOODIV v2 includes a robust time-calibrated phylogeny of all 210 tree species built from a published phylogenetic information complemented by the analysis of WOODIV v1 DNA-sequences, thus providing temporal context on species diversification.

## Methods

**Fig. 1:**
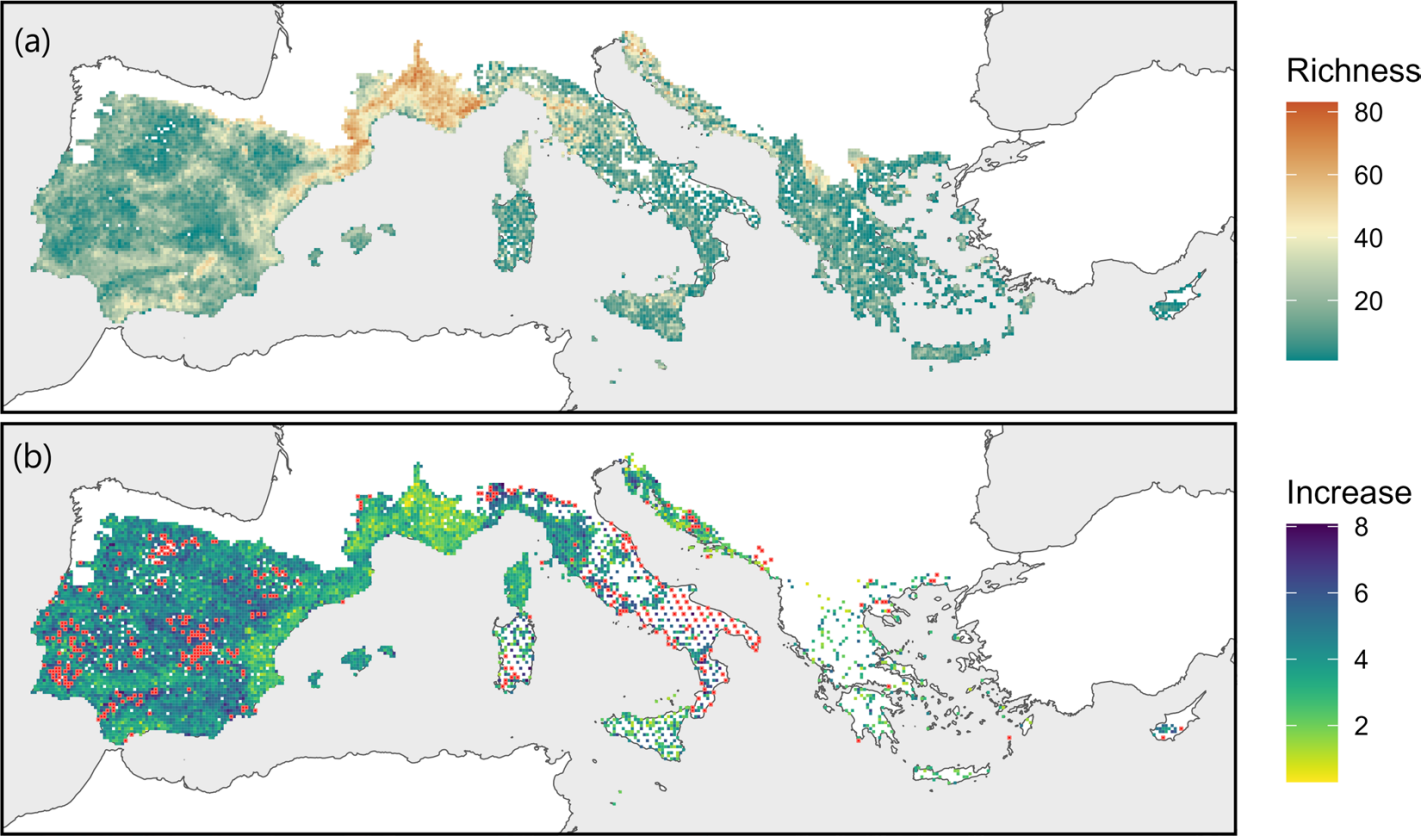
Geographic scope of the WOODIV database with spatial distribution of tree species occurrences, and comparison of the two versions of the WOODIV database. (**a**) Number of aggregated taxa at the gragg level (i.e., possible maximum = 206) within a 10 × 10 km grid cell (**b**) log-transformed percentage of increase in the number of aggregated taxa (gragg level) per 10 × 10 km grid cells between WOODIV v1 and v2; grid cells for which no data was available in v1 (therefore data increase cannot be calculated) but with data in v2 are displayed in red.

The geographic area covered by WOODIV v2 remains identical to the one covered by WOODIV v1 (Fig. 1a; all or part of the following countries and islands: Albania, Croatia, Cyprus, France, Greece, Italy, Malta, Montenegro, Portugal, Slovenia, Northern Macedonia, and Spain, including the Balearic archipelago, Corsica, Sardinia, Sicily, and Crete), so was the list of tree taxa considered (245 taxa - 210 species and 35 subspecies^11^). Two aggregation levels are available in the database to account for taxonomic resolution disparities between the different types of data and sources and taxonomic uncertainties around some closely related species groups. Subspecies can be aggregated at the species level (spagg, 210 species) and the following taxa can be aggregated at the species group level (gragg, 206 aggregated taxa): *Pinus uncinata* DC. and *P. mugo* Turra into *P. mugo* aggr., *Juniperus deltoides* R.P.Adams and *J. oxycedrus* L. into *J. oxycedrus* aggr. and *Alnus lusitanica* Vít, Douda & Mandák., *A. rohlenae* Vít, Douda & Mandák, and *A. glutinosa* (L.) Gaertn. into *A. glutinosa* aggr.

### Occurrence data

Sources of occurrence data considered in v1 were reviewed to determine whether updated datasets existed. New data were retrieved when available (Supplementary Table 1: updated sources indicated in bold; Supplementary Table 2) and spatially-aggregated at a resolution of 10 × 10 km in line with the INSPIRE compliant 10 × 10 km grid (SCR EPSG 3035) used in WOODIV v1. Geographical extent of the request from some sources considered in WOODIV v1 were extended to the whole study area (Supplementary Table 1: geographically extended sources indicated with “**”; Supplementary Table 2).

In addition, tree occurrence data for the 210 species (and 35 subspecies, totalling 245 taxa) were retrieved from three sources not considered in WOODIV v1 (Supplementary Table 1: new sources indicated with “*”; Supplementary Table 2), and spatially-aggregated at the 10 × 10 km resolution. As in the previous version, sources of occurrence data with a resolution coarser than 10 × 10 km were not considered. These new data sources include sPlotOpen^16^ (global vegetation plot data base), IFN (plot database of the French national inventory), and AFLIBER^17^ (Atlas of the Flora Iberica Database, covering Spain and Portugal).

Finally, WOODIV v2 provides tree occurrence data from intensive sampling campaigns carried out in spring-summer 2023 and 2024 (162 days). Sampling design corresponded to a 1/6 regular subsample of the 2,025 10 × 10 km grid cells covered at least by 25% of land occurring in continental Italy, Sicily and Sardinia. The sampling strategy has been designed to provide accurate information for the whole country (i.e., homogeneous sampling effort across administrative of biogeographic regions), especially when upscaled at coarser resolution (e.g., more than one 10 × 10 km grid with data per 50 × 50 km grid cell). Six experienced botanists prospected the 339 selected 10 × 10 km grid cells and recorded the occurrences of tree species and subspecies from the checklist.

WOODIV v2 increases spatial coverage of v1 by 4% (10,444 10 × 10 km grid cells with at least one occurrence record vs 10,042 in v1; Fig. 1b), particularly in Spain, France, Italy and in the Balkan area. Also, it allowed to obtain the only one European known location for *Tamarix passerinoides* Delile ex Desv.^18^, for which occurrence data were missing in v1. The WOODIV v2 database provides 269,313 records (unique combination of aggregated taxa at the gragg level, grid cell and source of data; 170,592 in v1) and 205,351 occurrences (unique combination of aggregated taxa (gragg level) and grid cell = removal of duplicate species (from different sources) within a grid cell; 145,954 in v1), corresponding to an increase between v1 and v2 by 57.9% and 40.7% respectively. The median increase in the number of aggregated taxa at gragg level per grid cell is 27.3%.

### Functional traits data

First, we updated traits data from WOODIV v1 for Specific Leaf Area (SLA), Stem Specific Density (StemSpecDens) and seed dry mass (SeedMass) for the 210 considered species (and 35 subspecies, totalling 245 taxa) using (i) data query from the TRY^19^ (https://www.try-db.org/) and BIEN^20^ (https://bien.nceas.ucsb.edu/bien/) repositories and (ii) new measurements from field work. (i) When available in TRY and BIEN, records original IDs, full references, methodological details (derived from traits codes for TRY data) and geographical coordinates converted to SCR EPSG 3035 were kept in order to easily access important contextual information. (ii) We took advantage of field work in Italy and southeastern France during the INTEGRADIV Biodiversa + project to collect samples to fill gaps in trait data remaining after the TRY and BIEN update. For specific leaf area, we followed Pérez-Harguindeguy *et al*.^21^ protocol. First, we collected 3–120 (depending on leaf area) mature, healthy and sunlit leaves, removed petioles and rachis and scanned them while they were fresh. Leaf area was determined using the FAMeLeS automatic pipeline^22^ and leaves were oven dried to weight dry mass to the nearest mg. For stem specific density, we collected wood cores from the main stem of c. 5 mm of diameter and c. 30 mm of length using an increment borer. Bark section was removed and actual length and diameter of the wood core were measured on the field using a 0.01 mm precision calliper to calculate the fresh volume (using the volume formula of a cylinder). Then, wood cores were oven dried and dry mass was measured to the nearest mg. For seed mass, we collected at least 100 mature seeds in each accession (c. 50 seeds in some accessions for species producing very big seeds such as *Quercus* sp. pl.), oven dried them and weighed the whole seeds lot to the nearest mg. The seed mass is then expressed as the dry mass of a single seed. For *Quercus* sp. pl. and *Prunus* sp. pl. we measured seed mass both including and excluding the pericarps and endocarps, respectively, to ensure relevant comparisons with other records. For all three traits, each record corresponds to the trait value at the individual level (from the same mother tree for seed mass). When possible, we sampled each species three times in different 10 × 10 km grid cells.

Besides the above-mentioned traits, WOODIV v2 includes 15 additional quantitative and categorical morpho-anatomical, reproductive and phenological traits. Categorical traits are rarely available in traits databases, but they have the advantage of being available for many species in botanical literature and can be easily used to compute distance based functional diversity indices^23^. Furthermore, the ecological significance of categorical leaf morphological traits to investigate macroecological patterns has been demonstrated^24,25^ and reproduction traits, including phenological characteristics, revealed independent dimensions within the plant economic spectrum^15^. We selected categorical traits which were widely available in national and regional floras and botanical literature for the list of considered tree taxa.

New morpho-anatomical traits encompass maximum tree height (HeightMax), leaf area (LeafArea), outline (LeafOutline), shape (LeafShape) and margin (LeafMargin). In this new version of the WOODIV database, we removed tree height gathered from TRY in v1 because this trait is very dependent on the individual age and is therefore hardly comparable across records and species. Instead, we collected maximum typical height recorded in botanical description from the main national and regional floras of the Euro-Mediterranean Basin (references in the Trait_sources file). Leaf area was obtained from TRY and BIEN repositories along with new measurements (cf. protocol for SLA measurement above). Also, we measured LeafArea from digitized herbarium specimens available online using the ImageJ software. Although area shrinkage in dry leaves, compared to fresh leaves, is very often negligible^26^, we flagged those records using the “method” field in the Trait_data file. Leaf outline, shape and margin were compiled based on expert knowledge, following Ellis *et al*.^27^. Leaf outline refers to the general outline of the leaf, i.e., either “entire”, “lobed” (apical sinus is incised by > 25% of the distance from the projection apex to the midvein) or “compound” (the leaf differentiates into several leaflets). Leaf shape indicates whether the leaf is “elliptic” (leaf blade length: width < 10: 1), “linear” (leaf blade length: width > 10: 1), “scaly” (very small scale-like leaf such as in *Tamarix* sp. pl., see Tavşanoğlu and Pausas (2018)^28^), “palmately_lobed”, “palmately_compound”, “pinnately_lobed”, “pinnately_compound”, i.e., whether leaves are lobed/compound following a palmate (major veins of lobes/leaflets arise from leaf base) or pinnate (major veins of lobes/leaflets are formed by coastal secondaries) pattern. Leaf margin indicates whether the leaf blade margin is “entire” or “toothed”.

To address reproductive strategies, we added in WOODIV v2: diaspore dispersal distance class (DispDist) and mode (DispMode), pollination mode (Pollination), and sexual system (SexSys). Diaspore dispersal distance class (from 2 to 6, with no human intervention) and mode (either “anemochory”, “dyszoochory”, “endozoochory”, “myrmecochory” or “unassisted”) were extracted from Lososová *et al*.^14^. For the 20 species for which the original dataset had no information, dispersal distance class and mode were assigned using Lososová *et al*.’ s decision tree. Pollination mode is either “abiotic” (wind mediated), “biotic” (insect mediated) or “mixte” (both wind and insect mediated). Sexual system is either “cosexuality” (hermaphrodite flowers), “monoecy” (separate male and female flowers on the same individual), “dioecy” (separate male and female flowers on different individuals) or “mixte” (refers to mixte syndromes like polygamy or androdioecy). Pollination mode and sexual system were obtained from floras and additional scientific literature.

Also, we added in WOODIV v2 new phenological traits: leaf phenology (LeafPheno) and blooming period traits. Leaf phenology indicates whether a species is either “evergreen”, “deciduous” or has an “intermediate” strategy (i.e., species qualified as semi-evergreen, semi-deciduous, or marcescent) and was obtained mainly from BROT2 database^28^ and floras. Blooming period start (BloomStart), end (BloomEnd) and position (BloomPosition, mean bloom month) are indicated by month position (from 1 for January to 12 for December) and breadth (BloomBreadth) indicates bloom duration (months). Blooming period start and end months were directly obtained from floras, while blooming period position and breadth were derived measurements.

All traits included in WOODIV v2 are described and sourced in Table 1.

**Table 1:** Summary of the 17 functional traits in WOODIV v2.

Trait name	Definition	Main sources
LeafShape*	Typical shape of the leaf, either “linear”, “palmately_lobed”, “palmately_compound”, “pinnately_lobed”, “pinnately_compound”, “elliptic”, or “scaly”	ined.
LeafMargin*	Margin of the leaf blade, either “entire” or “toothed”	ined.
LeafOutline*	Outline of the leaf, either “entire”, “lobed” or “compound”	ined.
LeafArea*	Area of the leaf [0.090–1,140 mm²]	TRY, BIEN, ined.
SLA	Specific leaf area [5 × 10^−6^ - 80 mm².mg^−1^]	TRY, BIEN, ined.
HeightMax*	Typical maximum tree height [0.7–50 m]	Floras
StemSpecDens	Stem specific density [0.090–1.140 g.cm^−3^]	TRY, BIEN, ined.
DispMode*	Diaspore dispersal mode, either “anemochory”, “dyszoochory”, “endozoochory”, “myrmecochory” or “unassisted”	Lososová *et al*., 2023
DispDist*	Diaspore dispersal distance classes, from “2” to “6”	Lososová *et al*., 2023
Pollination*	Pollination mode, either “abiotic”, “mixte” or “biotic”	Floras and additional literature
SexSys*	Sexual system, either “cosexuality”, “monoecy”, “dioecy” or “mixte”	Floras and additional literature
SeedMass	Seed dry mass [8 × 10^−3^ – 15,641 mg]	TRY, BIEN, ined.
LeafPheno*	Phenology of the leaves, either “deciduous”, “evergreen” or has an “intermediate” strategy (i.e., species qualified as semi-evergreen, semi-deciduous, or marcescent)	Floras, BROT2
BloomStart*	Starting month of the blooming period [1–12, month position]	Floras
BloomEnd*	Ending month of the blooming period [1–12, month position]	Floras
BloomPosition*	Average month of blooming [1–12.5, month position]	Floras
BloomBreadth*	Duration of the blooming period [1–8 months]	Floras

Stars “*” denote traits which are newly included in this version. When applicable, values range in the dataset along with units are indicated in brackets. ined. = unpublished data.

**Fig. 2:**
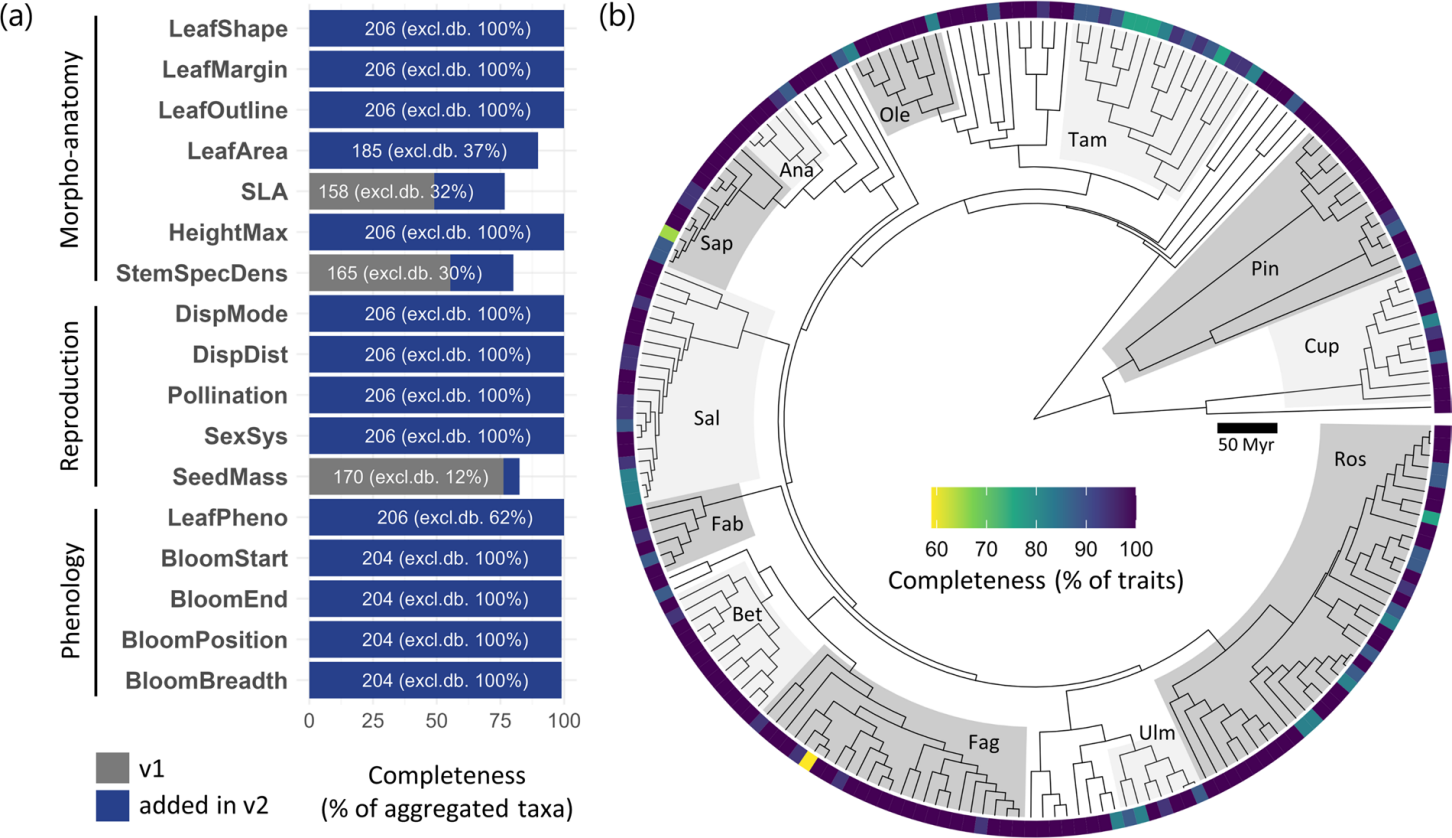
Traits data completeness in WOODIV v2 and time-calibrated phylogeny for taxa aggregated at the gragg level (n = 206): **(a)** percentage of aggregated taxa with at least one record across traits, data already present in v1 are displayed in grey, while blue parts depict data gain in v2. White numbers indicate the number of aggregated taxa (gragg level) for which trait value is available and the percentage in parenthesis (excl. db %) indicate the proportion of trait values by taxa which are not available in commonly used traits data bases (i.e., TRY, BIEN and BROT2). In **(b)**, traits completeness (percentage of traits with at least one record, for each taxon) is plotted against the new time-calibrated phylogeny at the gragg level and main families (with at least 5 species) are highlighted (Ros: Rosaceae; Ulm: Ulmaceae; Fag: Fagaceae; Bet: Betulaceae; Fab: Fabaceae; Sal: Salicaceae, Sap: Sapindaceae; Ana: Anacardiaceae; Ole: Oleaceae; Tam: Tamaricaceae; Pin: Pinaceae; Cup: Cupressaceae).

To compare data increase between versions 1 and 2, we computed relative increase as $\frac{{n}_{{v}_{2}}-{n}_{{v}_{1}}}{{n}_{{v}_{1}}}\times 100$, where ${n}_{{v}_{1}}$ and ${n}_{{v}_{2}}$ correspond to the number of unique combinations between a given trait and a given taxon at the gragg level, in version 1 and 2 respectively. WOODIV v2 allowed to substantially increase traits information for Euro-Mediterranean trees readily available in a data base (Fig. 2a): 3,348 traits × taxa unique combinations are available in v2, corresponding to an 800% increase relative to v1 (372 unique traits × taxa combinations, without considering tree height). Besides including more traits, taxonomic coverage increased as well, since all 206 aggregated taxa have traits information in v2 (versus 167 in v1, without considering tree height). Even with more traits considered, median traits completeness per gragg taxa (% of traits for which there is at least one recorded value for a given taxon) increased as well from 67% in v1 to 100% in v2. Also, out of the 3,348 traits × taxa (gragg level) unique combinations available in v2, 2,781 (i.e., 83%) are from new traits measurements and compilation from botanical literature, and thus not available in the commonly used traits databases TRY, BIEN and BROT2. Indeed, most existing plant traits data sets such as TRY and BIEN primarily focus on quantitative traits, and rarely on categorical ones. Traits and occurrence data availability for each of the 210 considered species (spagg level) is shown in Supplementary Table 3.

### Time-calibrated phylogeny

The chronogram for all 210 tree species considered in WOODIV was built following a three-step procedure. (i) First, we extracted the backbone phylogeny from the GBOTB.extended mega-phylogeny^29,30^, in which 148 species considered in WOODIV occurred. (ii) Then, the WOODIV v1 phylogenetic tree and published phylogenies were used for the topological placement in the backbone tree of 38 and 24 species, respectively (see Supplementary Table 4). (iii) Finally, the phylogenetic tree we obtained *via* step (i) and (ii) was calibrated to obtain a chronogram using the *chronos* function from the *ape* R package^31,32^, using a “discrete” rates model and a set of 32 secondary calibration points obtained from published works (see ages and references in Supplementary Table 5). We included as calibration points stem (SN) and crown nodes (CN) of Angiosperms and Gymnosperms, Eudicots (CN) and Core Eudicots (SN), Monocots (CN), Superarsterids (CN), and Malvids (SN and CN) to constrain diversification timing of the main clades. In addition, we included calibration points at lower taxonomic levels within Gymnosperms (CN of Cupressales order, Pinaceae and Cupressaceae families, *Abies* (both SN and CN), *Pinus*, and *Juniperus* genera) and Angiosperms (CN of Fabales, Fagales, Dipsacales and Rosales orders, Sapindaceae, Rhamnaceae, Anacardiaceae, Rosaceae and Ericaceae families, *Quercus* subgen. *Cerris*, *Acer*, *Salix*, *Rhamnus*, and *Pistacia* genera and SN of the *Cotoneaster* and *Crataegus* genera). The database includes a second time-calibrated phylogenetic tree pruned at the gragg aggregation level to match other data aggregated at this level (Fig. 2b).

## Data Records

**Fig. 3:**
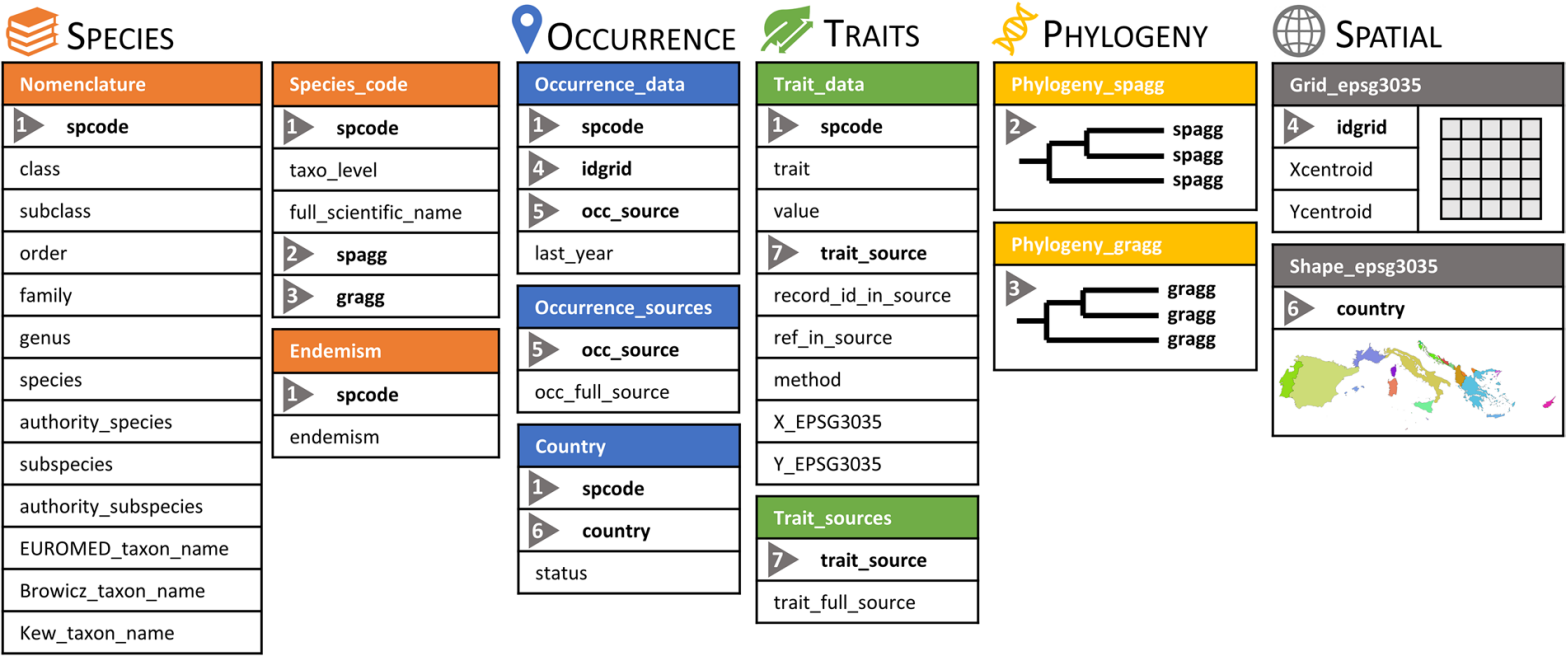
Structure of the WOODIV v2 database. Each block represents one table in the database. The files providing the information on species (in orange), occurrences (in blue), on traits (in green), on phylogeny (in yellow) and on spatial limits (in grey), can be linked via ‘spcode, ‘spagg’, ‘gragg’, ‘idgrid’, ‘country’, ‘occ_source’ and ‘trait_source’ keys. The definition and detailed information of all files and fields are described in Supplementary Tables 6, 7.

The data from WOODIV v2 are available on the figshare data repository 10.6084/m9.figshare.28513895^33^ and correspond to twelve files divided into five folders (Fig. 3), all named following the pattern “WOODIV_v2_filename.ext”. All comma-separated values (csv) files are UTF-8 encoded and in long table format.

The “SPECIES” folder includes three datasets in csv format (Supplementary Tables 6, 7): the “Species_code” file matches the species codes used in the WOODIV v2 database, the scientific name as defined by Médail *et al*.^11^ and the two aggregation levels at species level (spagg) and at group level (gragg); the “Nomenclature” file includes the nomenclature data of all taxa, from the order to the species or subspecies levels, and synonymous names if any; the “Endemism” file indicates which taxon is endemic following Médail *et al*.^11^ definition.

The “OCCURRENCE” folder includes three datasets in csv format (Supplementary Tables 6, 7). The “Occurrence_data” file includes all observed occurrences of taxa at the grain size of 10 × 10 km aligned with the INSPIRE LAEA grid, the code of the source from which the data was extracted and the most recent year at which the occurrence record was recorded in the grid cell when available; the “Occurrence_sources” file matches the source code to the full description of the source; the “Country” file shows whether the taxon is native or introduced in each country following Médail *et al*.^11^.

The “TRAITS” folder includes two datasets in csv format (Supplementary Tables 6, 7): the “Trait_data” table includes the functional traits values reported at the level of individual records with species codes (“spcode”), the code of the source from which they were extracted along with, when applicable, the record ID and reference within this database, methodological details and coordinates (SCR EPSG 3035) of the location where the measurement has been done; the “Trait_sources” table matches the source code to the full description of the source.

The “PHYLOGENY” folder includes two rooted time-calibrated phylogenetic trees in Newick format. The “Phylogeny_spagg” file includes the phylogenetic tree with the 210 species (aggregated taxa at spagg level) from the checklist. The “Phylogeny_gragg” file includes the phylogenetic tree with 206 aggregated taxa at gragg level.

The “SPATIAL” folder has two subfolders: the “Shape” subfolder includes a polygon shapefile (SCR EPSG 3035) delimiting the study area with country delimitations, while the “Grid” subfolder includes a polygon shapefile (SCR EPSG 3035) displaying the part of the INSPIRE LAEA grid that covers the study area.

## Technical Validation

All filters and checks implemented in Monnet *et al*.^7^ on WOODIV v1 to validate the data and to assure their reliability were implemented for WOODIV v2 when applicable. Moreover, the three data sources newly retrieved in WOODIV v2 (AFLIBER, sPlotOpen and IFN, Supplementary Table 1) are databases that already benefited from a process of cleaning and validation of occurrence data. The comparison of WOODIV v1 with the Atlas Flora Europaeae (AFE) have shown that overall, the v1 brought more occurrence information than missed. WOODIV v2 was compared to WOODIV v1 in order to ensure that no data were lost and to assess the increase in coverage offered by the v2 (Fig. 1b). This comparison showed that the WOODIV v2 successes in ameliorating the spatial coverage of the occurrence data, with a high number of 10 × 10 km grid cells with data records in v2 that were unsampled in v1 (n = 421; Fig. 1b, red cells), especially in Italy and Montenegro, countries identified in Monnet *et al*.^7^ as poorly sampled, but also in Spain and Croatia.

For traits data, contrary to the first version of the database, we did not filter out records from outside of the Euro-Mediterranean Basin, because, depending on the intended usage, one might be interested in keeping as much trait variation as possible. However, users can easily select traits records inside the Euro-Mediterranean range using records geographic coordinates and the Shape_epsg3035 file. Besides, we further checked traits data by comparing values in our data set to known ranges in Pérez-Harguindeguy *et al*.^21^ for specific leaf area, leaf area, stem specific density and seed mass.

**Fig. 4:**
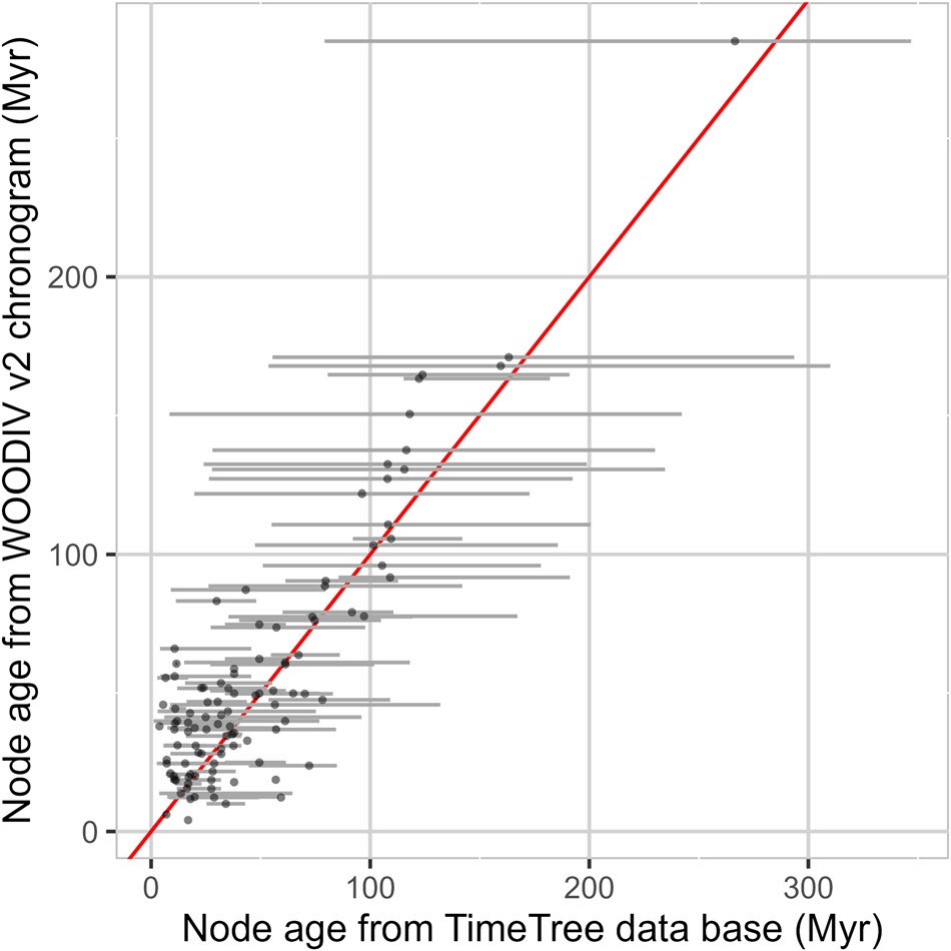
Divergence time (Myr) comparison between node age estimation in the WOODIV v2 chronogram (y-axis) and estimations from the literature extracted from the TimeTree database (x-axis, n = 141 nodes). Dots correspond to node age from a single estimation when only one record was available in TimeTree, or the median estimation when several records were available (in this case, the associated grey bars represent the range between the oldest and youngest estimates). The x = y reference line is displayed in red.

For the time-calibrated phylogenetic tree, we checked divergence times based on the comparison between node age estimation in the WOODIV v2 chronogram and estimations from the literature retrieved through the TimeTree database^34^ (of the 209 nodes in our phylogenetic tree at the “spagg” level, 141 (i.e., 67%) had at least one divergence time estimate recorded in the TimeTree database, totalling 1,256 estimates encompassing all families and genera, Fig. 4). Our estimates were + 10% older on average (interquartile range: −5.4% to 53.9%) and highly correlated (Pearson *r* = 0.93, *p-value* < 0.001) to the ones from TimeTree (single value, or median estimate for nodes with several records). When focusing on adjusted estimates from TimeTree (i.e., divergence times are reconciled across multiple phylogenies such that parent nodes are older than descendant nodes, data available for 107 nodes), the average error drops to + 0.8% (interquartile range: −15.5% to 44.4%) and correlation is still strong (*r* = 0.90, *p-value* < 0.001). Of the 99 nodes for which several divergence time estimations were available in TimeTree, 65% have divergence time estimate from WOODIV v2 lying between the minimum and maximum values in TimeTree, 28% have an older age than the oldest estimate from TimeTree and 7% have an younger age than the youngest estimate from TimeTree. Overall, and given the strong uncertainties existing in the literature, our time-calibrated phylogenetic trees fairly reflect diversification timing known for the taxa we considered.

## Usage Notes

DNA-sequences, phylograms and modelled occurrences provided by WOODIV v1 are not included in WOODIV v2, but are still available in the WOODIV v1 archive (10.6084/m9.figshare.13952897.v2). In any case, when using data from WOODIV v2, please cite both the present work and Monnet *et al*.^7^.

## Supplementary information

Supplementary Information and Tables

## Data Availability

The data from WOODIV v2 are available on the figshare data repository 10.6084/m9.figshare.28513895^33^.

## Code availability

All R codes used to produce the figures and to obtain the key metrics presented in this paper are available in Supplementary Information.
