# Supplementary material for: WOODIV v2, more occurrences, functional traits, and a time-calibrated phylogeny for Euro-Mediterranean trees
Source: Sci Data. 2025 Nov 6;12:1756. doi: 10.1038/s41597-025-06050-0 (PMC12592477; doi:10.1038/s41597-025-06050-0)
Supplement: Supplementary file 1 — Supplementary Information and Tables [file 41597_2025_6050_MOESM1_ESM.docx]

**Supplementary Information**

***Supplementary Table 1. Sources of the occurrence data considered in WOODIV v2.*** *Stars “*” denote sources which are newly included in this version. Names in bold denote datasets which have been updated between the two versions and double stars “**” datasets for which the geographical extent of the data retrieval was extended. Origin and citation of the sources are indicated in Supplementary Table 2. “Code v2” refers to the identifier of the source within the “source file” in the version 2 of the database, “Ref v2” to the reference number of the source in Supplementary Table 2; v1 code and ref are indicated, related to Monnet et al. (2021)); “Source name” refers to the Name of the dataset (ined. if unpublished) in v1 or v2. We indicated for each source the number of aggregated taxa at the gragg level (“Nb gragg”), the number of records (“Nb rec”), the contribution of the source records to the total WOODIV v2 database (“% rec”) and the number of 10 × 10km grid cells covered by the source (“Nb cells”).*

| **Ref v1** | **Code v1** | **Source name v1** | **Ref v2** | **Code v2** | **Source name v2** | **Nb gragg** | **Nb rec** | **Nb cells** | **% rec** |
| --- | --- | --- | --- | --- | --- | --- | --- | --- | --- |
| 1 | ALB | Distribution atlas of vascular plants in Albania | 1 | ALB | Distribution atlas of vascular plants in Albania | 103 | 2873 | 291 | 1,07 |
| 2 | **CRO** | **Flora Croatica Database** | 2a | CRO | Flora Croatica Database | 23 | 346 | 174 | 0,13 |
|  |  |  | 2b | FCD | Flora Croatica Database v2 | 104 | 7472 | 333 | 2,77 |
| 3 | CYP | ined. | 3 | CYP | ined. | 6 | 38 | 27 | 0,01 |
| 4 | EUR_EUFORGEN | EUFGIS Genetic Conservation Units | 4 | EUR_EUFORGEN | EUFGIS Genetic Conservation Units | 23 | 173 | 148 | 0,06 |
| 5 | EUR_GBIF | GBIF | 5 | EUR_GBIF | GBIF | 22 | 81 | 33 | 0,03 |
| 6 | **FRA_CBNA** | **Conservatoire botanique national alpin (CBNA)** | 6a | FRA_CBNA | Conservatoire botanique national alpin (CBNA) | 94 | 2973 | 86 | 1,10 |
| 7 | **FRA_CBNC** | **Conservatoire botanique national de Corse (CBNC)** | 7a | FRA_CBNC | Conservatoire botanique national de Corse (CBNC) | 24 | 175 | 84 | 0,06 |
| 10 | **FRA_SIL** | **Conservatoire botanique national méditerranéen de Porquerolles (CBNMed)** | 10a | FRA_SIL | Conservatoire botanique national méditerranéen de Porquerolles (CBNMed) | 96 | 1497 | 514 | 0,56 |
|  |  |  | 6-7-10b | SIMETHIS | Simethis (CBNA, CBNC, CBNMed) | 117 | 29490 | 712 | 10,95 |
| 8 | **FRA_CBNMC** | **Conservatoire botanique national du Massif Central (CBNMC)** | 8a | FRA_CBNMC | Conservatoire botanique national du Massif Central (CBNMC) | 47 | 247 | 52 | 0,09 |
|  |  |  | 8b | SINP_AURA | SINP Auvergne Rhône-Alpes | 100 | 6128 | 122 | 2,28 |
| 9 | **FRA_IGN** | **IGN Inventaire Forestier** | 9a | FRA_IGN | IGN Inventaire Forestier | 28 | 215 | 171 | 0,08 |
|  |  |  | 9b* | IFN | IGN Inventaire Forestier | 109 | 20663 | 814 | 7,67 |
| 11 | GRE | Flora Hellenica Database | 11 | GRE | Flora Hellenica Database | 139 | 17612 | 1616 | 6,54 |
| 12 | **IBE** | **GBIF** | 12a | IBE | GBIF | 105 | 17667 | 4415 | 6,56 |
|  |  |  | 12b | FLORAON | Flora-On | 70 | 3511 | 744 | 1,30 |
|  |  |  | 12c* | AFLIBER | Atlas Flora Iberica | 98 | 84378 | 5058 | 31,33 |
| 13 | ISL | Tentamen Florae Aeolicae | 13 | ISL | Tentamen Florae Aeolicae | 46 | 246 | 21 | 0,09 |
| 14 | ITA_CNR | CNR data | 14 | ITA_CNR | CNR data | 5 | 184 | 181 | 0,07 |
| 15 | **ITA_EUFOREST** | **EU-Forest (for Italy)** |  |  |  |  |  |  | 0,00 |
|  |  |  | 15b** | EUFOREST | EU-Forest (for Europe) | 107 | 24875 | 5957 | 9,24 |
| 16 | ITA_INFI | INFC 2015 | 16 | ITA_INFI | INFC 2015 | 65 | 1089 | 610 | 0,40 |
| 17 | ITA_VEG | VegItaly | 17 | ITA_VEG | VegItaly | 93 | 5765 | 681 | 2,14 |
| 18 | **ITA_WPB** | **WikiPlantBase # Liguria, # Toscana, # Sardegna, # Sicily** | 18a | ITA_WPB | WikiPlantBase # Liguria, # Toscana, # Sardegna, # Sicily | 73 | 850 | 308 | 0,32 |
|  |  |  | 18b** | ITA_WPB2 | WikiPlantBase #Italy | 118 | 13892 | 953 | 5,16 |
| 19 | MAC | ined. | 19 | MAC | ined. | 99 | 3396 | 88 | 1,26 |
| 20 | MAL | ined. | 20 | MAL | ined. | 39 | 154 | 7 | 0,06 |
| 21 | MONTE | ined. | 21 | MONTE | ined. | 69 | 731 | 25 | 0,27 |
| 22 | SIC | ined. | 22 | SIC | ined. | 95 | 4194 | 338 | 1,56 |
| 23 | SLO | Atlas of Flora of Slovenia | 23 | SLO | Atlas of Flora of Slovenia | 87 | 1260 | 33 | 0,47 |
| 24 |  |  | 24* | SPLOT | sPlot Open | 148 | 9185 | 1268 | 3,41 |
| 25 |  |  | 25* | WOODIV_V2 | ined. | 134 | 7953 | 339 | 2,95 |

***Supplementary Table 2. References of the sources of the occurrence records****. Ref is the reference number to Supplementary Table 1.*

| **Ref** | **Code v2** | **Full Source** |
| --- | --- | --- |
| 1 | ALB | Barina, Zoltán (2017) Distribution atlas of vascular plants in Albania. Hungarian Natural History Museum, Budapest. 492 pp. |
| 2a | CRO | Nikolić T. ed. (2015-2024): Flora Croatica Database (URL: https://hirc.botanic.hr/fcd/). Faculty of Science, University of Zagreb (Accessed the 08/09/2016) |
| 2b | FCD | Nikolić T., Bogdanović S., Vuković N., Šegota V. eds. (2025) Flora Croatica Database (URL: https://hirc.botanic.hr/fcd/). Faculty of Science, University of Zagreb Accessed the 19/01/2025. |
| 3 | CYP | Charalambos S. Christodolou, personal communication. Accessed the 27/02/2018 |
| 4 | EUR_EUFORGEN | Genetic Conservation Units from the EUFGIS (http://portal.eufgis.org) provided by the European Forest Genetic Resources Programme (EUFORGEN) on 04/01/2017 |
| 5 | EUR_GBIF | For Cyprus only, Serra-Diaz et al. (2017) Big data of tree species distributions: how big and how good? For. Ecosyst. 4, 30. Accessed the 20/10/2017 |
| 6a | FRA_CBNA | Conservatoire botanique national Alpin (CBNA). Accessed the 28/03/2017 |
| 7a | FRA_CBNC | Conservatoire botanique national de Corse (CBNC). Accessed the 09/11/2017 |
| 10a | FRA_SIL | Système d’Information et de Localisation des Espèces Natives et Envahissantes (SILENE) (http://flore.silene.eu). Accessed the 21/10/2016 |
| 6-7-10b | SIMETHIS | Conservatoire Botanique National Méditerranéen, Conservatoire Botanique National Alpin, Conservatoire Botanique National de Corse. SIMETHIS. Accessed the 03/02/2025. |
| 8a | FRA_CBNMC | Conservatoire botanique national du Massif central. 14 février 2017 < CHLORIS® système d’information dédié à la flore sauvage et aux végétations du Massif central > |
| 8b | SINP_AURA | Observatoire de la Biodiversité en Auvergne-Rhône-Alpes, 2021. SINP AURA. Accessed the 24/01/2025. |
| 9a | FRA_IGN | IGN Inventaire forestier (http:/inventaire-forestier.ign.fr). Accessed the 05/04/2016 |
| 9b | IFN | IGN Inventaire forestier (http:/inventaire-forestier.ign.fr). Accessed the 08/01/2025. |
| 11 | GRE | Flora Hellenica Database (personal communication, prof. A. Strid, 2019), Dimopoulos P, Raus T, Strid A (ed.) (2018) Flora of Greece web. Vascular Plants of Greece. An Annotated Checklist. Version II (June 2018). Published at: http://portal.cybertaxonomy.org/flora-greece/, accessed 20 August 2018, Dimopoulos P, Raus T, Bergmeier E, et al (2013) Vascular plants of Greece: an annotated checklist. – Berlin: Botanischer Garten und Botanisches Museum Berlin-Dahlem, Freie Universität Berlin, Athens: Hellenic Botanical Society. Englera, 31: 1-370 |
| 12a | IBE | GBIF.org, Flora Iberica, Castroviejo, S. (coord. gen.). 1986-2012. Flora iberica 1-8, 10-15, 17-18, 21. Real Jardín Botánico, CSIC, Madrid., accessed with Anthos (http://www.anthos.es), Flora-On: Flora de Portugal Interactiva. (2014). Sociedade Portuguesa de Botânica. www.flora-on.pt. Consulta efectuada em 24-3-2020. |
| 12b | FLORAON | Flora-On: Flora de Portugal Interactiva. (2025). Sociedade Portuguesa de Botânica. www.flora-on.pt. Consulta efectuada em 08/01/2025. |
| 12c | AFLIBER | Ramos-Gutiérrez et al., 2021. Atlas of the vascular flora of the Iberian Peninsula biodiversity hotspot (AFLIBER). Global Ecology and Biogeography 30, 1951-1957. https://doi.org/10.1111/geb.13363. Data base accessed the 22/05/2023. |
| 13 | ISL | Pasta S., La Rosa A., Pavon D., Lo Cascio P., Médail F. Tentamen Florae Aeolicae: A critical checklist of the vascular plants of the Aeolian Islands (Sicily, Italy). in prep. |
| 14 | ITA_CNR | Data from the National Research Council (Italy), For Abies alba: Piotti A., Leonarduzzi C., Postolache D., Bagnoli F., Spanu I., Brousseau L., Urbinati C., Leonardi S., Vendramin G.G. (2017). Unexpected scenarios from Mediterranean refugial areas: disentangling complex demographic dynamics along the Apennine distribution of silver fir. Journal of Biogeography, 44(7), 1547-1558., For Fagus sylvatica: Bagnoli F., Piotti A., Vendramin G.G., personal communication, For Pinus halepensis: Gómez A, Vendramin G.G., González?Martínez S.C., Alía R. (2005). Genetic diversity and differentiation of two Mediterranean pines (Pinus halepensis Mill. and Pinus pinaster Ait.) along a latitudinal cline using chloroplast microsatellite markers. Diversity and Distribution, 11(3): 257-263., Bucci G., Anzidei M., Madaghiele A., Vendramin G.G. (1998). Detection of haplotypic variation and natural hybridization in halepensis?complex pine species using chloroplast simple sequence repeat (SSR) markers. Molecular Ecology, 7(12): 1633-1643., For Pinus heldreichii: Bagnoli F., Piotti A., Vendramin G.G., personal comunication, For Quercus cerris: Bagnoli F., Tsuda Y., Fineschi S., Bruschi P., Magri D., Zhelev P., Paule L., Simeone M.C., González-Martínez S.C. & Vendramin G.G. (2016) Combining molecular and fossil data to infer demographic history of Quercus cerris: insights on European eastern glacial refugia. Journal of Biogeography, 43, 679-690. |
| 15b | EUFOREST | Mauri et al., 2017. EU-Forest, a high-resolution tree occurrence dataset for Europe. Scientific Data, 160123. http://dx.doi.org/10.1038/sdata.2016.123. Data base accessed via https://doi.org/10.6084/m9.figshare.c.3288407.v1 the 12/05/2023. |
| 16 | ITA_INFI | Terzo inventario forestale nazionale (INFC2015) https://inventarioforestale.org/it |
| 17 | ITA_VEG | www.vegitaly.it, Lucarini D., Gigante D., Landucci F., Panfili E., Venanzoni R. 2015. The anArchive taxonomic Checklist for Italian botanical data banking and vegetation analysis: theoretical basis and advantages. Plant Biosyst., 149(6): 958-965. doi: 10.1080/11263504.2014.984010, Gigante D., Acosta A.T.R., Agrillo E., Attorre F., Cambria V.M., Casavecchia S., Chiarucci A., Del Vico E., De Sanctis M., Facioni L., Geri F., Guarino R., S. Landi, Landucci F., Lucarini D., Panfili E., Pesaresi S., Prisco I., Rosati L., Spada F., Venanzoni R., 2012. VegItaly: Technical features, crucial issues and some solutions. Plant Sociology, 49(2): 71-79. doi: 10.7338/pls2012492/05, Landucci F., Acosta A.T.R., Agrillo E., Attorre F., Biondi E., Cambria V.M., Chiarucci A., Del Vico E., De Sanctis M., Facioni L., Geri F., Gigante D., Guarino R., S. Landi, Lucarini D., Panfili E., Pesaresi S., Prisco I., Rosati L., Spada F., Venanzoni R., 2012. VegItaly: The Italian collaborative project for a national vegetation database. Plant Biosyst., 146(4): 756-763. doi: 10.1080/11263504.2012.740093, Venanzoni R., Landucci F., Panfili E., Gigante D., 2012. Toward an Italian national vegetation database: VegItaly. In: Dengler, J., Oldeland, J., Jansen, F., Chytry?, M., Ewald, J., Finckh, M., Glöckler, F., Lopez-Gonzalez, G., Peet, R.K., Schaminée, J.H.J. [Eds.]: Vegetation databases for the 21st century. Biodiversity & Ecology, 4: 185-190. ISSN: 1613-9801 |
| 18a | ITA_WPB | GBIF.org (21st June 2019) GBIF Occurrence Download https://doi.org/10.15468/dl.jnctii, GBIF.org (21st June 2019) GBIF Occurrence Download https://doi.org/10.15468/dl.5s23qb, GBIF.org (21st June 2019) GBIF Occurrence Download https://doi.org/10.15468/dl.seopf2, GBIF.org (27th June 2019) GBIF Occurrence Download https://doi.org/10.15468/dl.fd3wnl |
| 18b** | ITA_WPB2 | Peruzzi L. et al. (ed). Wikiplantbase #Italia (https://bot.biologia.unipi.it/wpb/italia). Accessed the 04/08/2024. |
| 19 | MAC | V. Matevski, ined. |
| 20 | MAL | Stephen Mifsud, ined. |
| 21 | MONTE | Médail & Monnet, ined. |
| 22 | SIC | Gianniantonio Domina, ined. |
| 23 | SLO | N Jogan, T Bacic, B Frajman, I Leskovar, D Naglic, A Podobnik, B Rozman, S Strguljc-Krajšek, B Trcak, 2001. Materials for the Atlas of Flora of Slovenia, Center za kartiranje favne in flore. Miklavz na Dravskem polju |
| 24* | SPLOT | Sabatini et al., 2021. sPlotOpen – An environmentally balanced, open-access, global dataset of vegetation plots. Global Ecology and Biogeography 30, 1740-1764. https://doi.org/10.1111/geb.13346. Data base accessed via https://idata.idiv.de/ddm/Data/ShowData/3474?version=76 the 26/04/2023. |
| 25* | WOODIV_V2 | WOODIV v2 data paper |

***Supplementary Table 3. Occurrence and traits data availability for the whole 210 tree species (spagg level).***

| Species (spagg) | Occ | Functional traits | | | | | | | | | | | | | | | | |
| --- | --- | --- | --- | --- | --- | --- | --- | --- | --- | --- | --- | --- | --- | --- | --- | --- | --- | --- |
|  |  | BloomBreadth | BloomEnd | BloomPosition | BloomStart | DispDist | DispMode | HeightMax | LeafArea | LeafMargin | LeafOutline | LeafPheno | LeafShape | Pollination | SeedMass | SexSys | SLA | StemSpecDens |
| Abies alba (AALB) | 1 | 1 | 1 | 1 | 1 | 1 | 1 | 1 | 1 | 1 | 1 | 1 | 1 | 1 | 1 | 1 | 1 | 1 |
| Abies borisii-regis (ABOR) | 1 | 1 | 1 | 1 | 1 | 1 | 1 | 1 | 1 | 1 | 1 | 1 | 1 | 1 | 1 | 1 | NA | NA |
| Abies cephalonica (ACEP) | 1 | 1 | 1 | 1 | 1 | 1 | 1 | 1 | 1 | 1 | 1 | 1 | 1 | 1 | 1 | 1 | 1 | 1 |
| Abies nebrodensis (ANEB) | 1 | 1 | 1 | 1 | 1 | 1 | 1 | 1 | 1 | 1 | 1 | 1 | 1 | 1 | NA | 1 | 1 | 1 |
| Abies pinsapo (APIN) | 1 | 1 | 1 | 1 | 1 | 1 | 1 | 1 | 1 | 1 | 1 | 1 | 1 | 1 | 1 | 1 | 1 | 1 |
| Acer campestre (ACAM) | 1 | 1 | 1 | 1 | 1 | 1 | 1 | 1 | 1 | 1 | 1 | 1 | 1 | 1 | 1 | 1 | 1 | 1 |
| Acer cappadocicum (ACAP) | 1 | 1 | 1 | 1 | 1 | 1 | 1 | 1 | 1 | 1 | 1 | 1 | 1 | 1 | 1 | 1 | 1 | 1 |
| Acer heldreichii (AHEL) | 1 | NA | NA | NA | NA | 1 | 1 | 1 | 1 | 1 | 1 | 1 | 1 | 1 | 1 | 1 | NA | NA |
| Acer hyrcanum (AHYR) | 1 | 1 | 1 | 1 | 1 | 1 | 1 | 1 | 1 | 1 | 1 | 1 | 1 | 1 | 1 | 1 | NA | NA |
| Acer monspessulanum (AMON) | 1 | 1 | 1 | 1 | 1 | 1 | 1 | 1 | 1 | 1 | 1 | 1 | 1 | 1 | 1 | 1 | 1 | 1 |
| Acer obtusifolium (AOBT) | 1 | 1 | 1 | 1 | 1 | 1 | 1 | 1 | 1 | 1 | 1 | 1 | 1 | 1 | 1 | 1 | NA | 1 |
| Acer opalus (AOPA) | 1 | 1 | 1 | 1 | 1 | 1 | 1 | 1 | 1 | 1 | 1 | 1 | 1 | 1 | 1 | 1 | 1 | 1 |
| Acer platanoides (APLA) | 1 | 1 | 1 | 1 | 1 | 1 | 1 | 1 | 1 | 1 | 1 | 1 | 1 | 1 | 1 | 1 | 1 | 1 |
| Acer pseudoplatanus (APSE) | 1 | 1 | 1 | 1 | 1 | 1 | 1 | 1 | 1 | 1 | 1 | 1 | 1 | 1 | 1 | 1 | 1 | 1 |
| Acer sempervirens (ASEM) | 1 | 1 | 1 | 1 | 1 | 1 | 1 | 1 | 1 | 1 | 1 | 1 | 1 | 1 | 1 | 1 | NA | NA |
| Acer tataricum (ATAT) | 1 | 1 | 1 | 1 | 1 | 1 | 1 | 1 | 1 | 1 | 1 | 1 | 1 | 1 | 1 | 1 | 1 | NA |
| Aesculus hippocastanum (AHIP) | 1 | 1 | 1 | 1 | 1 | 1 | 1 | 1 | 1 | 1 | 1 | 1 | 1 | 1 | 1 | 1 | 1 | 1 |
| Alnus cordata (ACOR) | 1 | 1 | 1 | 1 | 1 | 1 | 1 | 1 | 1 | 1 | 1 | 1 | 1 | 1 | 1 | 1 | 1 | 1 |
| Alnus glutinosa (AGLU) | 1 | 1 | 1 | 1 | 1 | 1 | 1 | 1 | 1 | 1 | 1 | 1 | 1 | 1 | 1 | 1 | 1 | 1 |
| Alnus incana (AINC) | 1 | 1 | 1 | 1 | 1 | 1 | 1 | 1 | 1 | 1 | 1 | 1 | 1 | 1 | 1 | 1 | 1 | 1 |
| Alnus lusitanica (ALUS) | NA | 1 | 1 | 1 | 1 | 1 | 1 | 1 | NA | 1 | 1 | NA | 1 | NA | NA | 1 | NA | NA |
| Alnus orientalis (AORI) | 1 | 1 | 1 | 1 | 1 | 1 | 1 | 1 | 1 | 1 | 1 | 1 | 1 | 1 | 1 | 1 | NA | 1 |
| Alnus rohlenae (AROH) | 1 | 1 | 1 | 1 | 1 | 1 | 1 | 1 | NA | 1 | 1 | NA | 1 | NA | NA | 1 | NA | NA |
| Alnus viridis (AVIR) | 1 | 1 | 1 | 1 | 1 | 1 | 1 | 1 | 1 | 1 | 1 | 1 | 1 | 1 | 1 | 1 | 1 | 1 |
| Arbutus andrachne (AAND) | 1 | 1 | 1 | 1 | 1 | 1 | 1 | 1 | 1 | 1 | 1 | 1 | 1 | 1 | 1 | 1 | 1 | 1 |
| Arbutus unedo (AUNE) | 1 | 1 | 1 | 1 | 1 | 1 | 1 | 1 | 1 | 1 | 1 | 1 | 1 | 1 | 1 | 1 | 1 | 1 |
| Betula pendula (BPEN) | 1 | 1 | 1 | 1 | 1 | 1 | 1 | 1 | 1 | 1 | 1 | 1 | 1 | 1 | 1 | 1 | 1 | 1 |
| Betula pubescens (BPUB) | 1 | 1 | 1 | 1 | 1 | 1 | 1 | 1 | 1 | 1 | 1 | 1 | 1 | 1 | 1 | 1 | 1 | 1 |
| Buxus balearica (BBAL) | 1 | 1 | 1 | 1 | 1 | 1 | 1 | 1 | 1 | 1 | 1 | 1 | 1 | 1 | 1 | 1 | 1 | 1 |
| Buxus sempervirens (BSEM) | 1 | 1 | 1 | 1 | 1 | 1 | 1 | 1 | 1 | 1 | 1 | 1 | 1 | 1 | 1 | 1 | 1 | 1 |
| Carpinus betulus (CBET) | 1 | 1 | 1 | 1 | 1 | 1 | 1 | 1 | 1 | 1 | 1 | 1 | 1 | 1 | 1 | 1 | 1 | 1 |
| Carpinus orientalis (CAOR) | 1 | 1 | 1 | 1 | 1 | 1 | 1 | 1 | 1 | 1 | 1 | 1 | 1 | 1 | 1 | 1 | 1 | 1 |
| Castanea sativa (CSAT) | 1 | 1 | 1 | 1 | 1 | 1 | 1 | 1 | 1 | 1 | 1 | 1 | 1 | 1 | 1 | 1 | 1 | 1 |
| Cedrus libani (CLIB) | 1 | 1 | 1 | 1 | 1 | 1 | 1 | 1 | NA | 1 | 1 | 1 | 1 | 1 | 1 | 1 | NA | 1 |
| Celtis australis (CAUS) | 1 | 1 | 1 | 1 | 1 | 1 | 1 | 1 | 1 | 1 | 1 | 1 | 1 | 1 | 1 | 1 | 1 | 1 |
| Celtis planchoniana (CPLA) | 1 | 1 | 1 | 1 | 1 | 1 | 1 | 1 | NA | 1 | 1 | 1 | 1 | 1 | 1 | 1 | NA | NA |
| Celtis tournefortii (CTOU) | 1 | 1 | 1 | 1 | 1 | 1 | 1 | 1 | 1 | 1 | 1 | 1 | 1 | 1 | 1 | 1 | 1 | 1 |
| Ceratonia siliqua (CSIA) | 1 | 1 | 1 | 1 | 1 | 1 | 1 | 1 | 1 | 1 | 1 | 1 | 1 | 1 | 1 | 1 | 1 | 1 |
| Cercis siliquastrum (CSIM) | 1 | 1 | 1 | 1 | 1 | 1 | 1 | 1 | 1 | 1 | 1 | 1 | 1 | 1 | 1 | 1 | 1 | 1 |
| Chamaerops humilis (CHUM) | 1 | 1 | 1 | 1 | 1 | 1 | 1 | 1 | 1 | 1 | 1 | 1 | 1 | 1 | 1 | 1 | 1 | 1 |
| Cotinus coggygria (CCOG) | 1 | 1 | 1 | 1 | 1 | 1 | 1 | 1 | 1 | 1 | 1 | 1 | 1 | 1 | 1 | 1 | 1 | 1 |
| Corylus avellana (CAVE) | 1 | 1 | 1 | 1 | 1 | 1 | 1 | 1 | 1 | 1 | 1 | 1 | 1 | 1 | 1 | 1 | 1 | 1 |
| Corylus colurna (CCOL) | 1 | 1 | 1 | 1 | 1 | 1 | 1 | 1 | 1 | 1 | 1 | 1 | 1 | 1 | 1 | 1 | 1 | 1 |
| Cotoneaster granatensis (CGRA) | 1 | 1 | 1 | 1 | 1 | 1 | 1 | 1 | 1 | 1 | 1 | 1 | 1 | 1 | 1 | 1 | NA | NA |
| Crataegus azarolus (CAZA) | 1 | 1 | 1 | 1 | 1 | 1 | 1 | 1 | 1 | 1 | 1 | 1 | 1 | 1 | 1 | 1 | 1 | 1 |
| Crataegus heldreichii (CHEL) | 1 | 1 | 1 | 1 | 1 | 1 | 1 | 1 | 1 | 1 | 1 | 1 | 1 | 1 | NA | 1 | NA | NA |
| Crataegus laciniata (CLAC) | 1 | 1 | 1 | 1 | 1 | 1 | 1 | 1 | 1 | 1 | 1 | 1 | 1 | 1 | 1 | 1 | 1 | 1 |
| Crataegus laevigata (CLAE) | 1 | 1 | 1 | 1 | 1 | 1 | 1 | 1 | 1 | 1 | 1 | 1 | 1 | 1 | 1 | 1 | 1 | 1 |
| Crataegus monogyna (CMON) | 1 | 1 | 1 | 1 | 1 | 1 | 1 | 1 | 1 | 1 | 1 | 1 | 1 | 1 | 1 | 1 | 1 | 1 |
| Crataegus nevadensis (CNEV) | 1 | 1 | 1 | 1 | 1 | 1 | 1 | 1 | 1 | 1 | 1 | 1 | 1 | 1 | NA | 1 | NA | NA |
| Crataegus orientalis (CROR) | 1 | 1 | 1 | 1 | 1 | 1 | 1 | 1 | 1 | 1 | 1 | 1 | 1 | 1 | 1 | 1 | NA | NA |
| Crataegus pentagyna (CPEN) | 1 | 1 | 1 | 1 | 1 | 1 | 1 | 1 | 1 | 1 | 1 | 1 | 1 | 1 | 1 | 1 | 1 | 1 |
| Crataegus pycnoloba (CPYC) | 1 | 1 | 1 | 1 | 1 | 1 | 1 | 1 | 1 | 1 | 1 | 1 | 1 | 1 | NA | 1 | NA | NA |
| Cupressus sempervirens (CSEM) | 1 | 1 | 1 | 1 | 1 | 1 | 1 | 1 | 1 | 1 | 1 | 1 | 1 | 1 | 1 | 1 | 1 | 1 |
| Cytisus aeolicus (CAEO) | 1 | 1 | 1 | 1 | 1 | 1 | 1 | 1 | 1 | 1 | 1 | 1 | 1 | 1 | 1 | 1 | 1 | NA |
| Erica arborea (EARB) | 1 | 1 | 1 | 1 | 1 | 1 | 1 | 1 | 1 | 1 | 1 | 1 | 1 | 1 | 1 | 1 | 1 | 1 |
| Fagus orientalis (FORI) | 1 | 1 | 1 | 1 | 1 | 1 | 1 | 1 | 1 | 1 | 1 | 1 | 1 | 1 | 1 | 1 | NA | 1 |
| Fagus sylvatica (FSYL) | 1 | 1 | 1 | 1 | 1 | 1 | 1 | 1 | 1 | 1 | 1 | 1 | 1 | 1 | 1 | 1 | 1 | 1 |
| Ficus carica (FCAR) | 1 | 1 | 1 | 1 | 1 | 1 | 1 | 1 | 1 | 1 | 1 | 1 | 1 | 1 | 1 | 1 | 1 | 1 |
| Fontanesia philliraeoides (FPHI) | 1 | 1 | 1 | 1 | 1 | 1 | 1 | 1 | NA | 1 | 1 | 1 | 1 | 1 | 1 | 1 | NA | NA |
| Frangula alnus (FALN) | 1 | 1 | 1 | 1 | 1 | 1 | 1 | 1 | 1 | 1 | 1 | 1 | 1 | 1 | 1 | 1 | 1 | 1 |
| Fraxinus angustifolia (FANG) | 1 | 1 | 1 | 1 | 1 | 1 | 1 | 1 | 1 | 1 | 1 | 1 | 1 | 1 | 1 | 1 | 1 | 1 |
| Fraxinus excelsior (FEXC) | 1 | 1 | 1 | 1 | 1 | 1 | 1 | 1 | 1 | 1 | 1 | 1 | 1 | 1 | 1 | 1 | 1 | 1 |
| Fraxinus ornus (FORN) | 1 | 1 | 1 | 1 | 1 | 1 | 1 | 1 | 1 | 1 | 1 | 1 | 1 | 1 | 1 | 1 | 1 | 1 |
| Fraxinus pallisae (FPAL) | 1 | 1 | 1 | 1 | 1 | 1 | 1 | 1 | NA | 1 | 1 | 1 | 1 | 1 | 1 | 1 | NA | NA |
| Genista etnensis (GETN) | 1 | 1 | 1 | 1 | 1 | 1 | 1 | 1 | NA | 1 | 1 | 1 | 1 | 1 | 1 | 1 | NA | 1 |
| Genista tyrrhena (GTYR) | 1 | 1 | 1 | 1 | 1 | 1 | 1 | 1 | 1 | 1 | 1 | 1 | 1 | 1 | 1 | 1 | 1 | 1 |
| Ilex aquifolium (IAQU) | 1 | 1 | 1 | 1 | 1 | 1 | 1 | 1 | 1 | 1 | 1 | 1 | 1 | 1 | 1 | 1 | 1 | 1 |
| Juglans regia (JREG) | 1 | 1 | 1 | 1 | 1 | 1 | 1 | 1 | 1 | 1 | 1 | 1 | 1 | 1 | 1 | 1 | 1 | 1 |
| Juniperus communis (JCOM) | 1 | 1 | 1 | 1 | 1 | 1 | 1 | 1 | 1 | 1 | 1 | 1 | 1 | 1 | 1 | 1 | 1 | 1 |
| Juniperus deltoides (JDEL) | 1 | NA | NA | NA | NA | 1 | 1 | NA | NA | 1 | 1 | NA | 1 | 1 | 1 | 1 | NA | NA |
| Juniperus drupacea (JDRU) | 1 | 1 | 1 | 1 | 1 | 1 | 1 | 1 | 1 | 1 | 1 | 1 | 1 | 1 | NA | 1 | NA | NA |
| Juniperus excelsa (JEXC) | 1 | 1 | 1 | 1 | 1 | 1 | 1 | 1 | 1 | 1 | 1 | 1 | 1 | 1 | 1 | 1 | NA | 1 |
| Juniperus foetidissima (JFOE) | 1 | 1 | 1 | 1 | 1 | 1 | 1 | 1 | NA | 1 | 1 | 1 | 1 | 1 | 1 | 1 | NA | 1 |
| Juniperus macrocarpa (JMAC) | 1 | 1 | 1 | 1 | 1 | 1 | 1 | 1 | 1 | 1 | 1 | 1 | 1 | 1 | 1 | 1 | 1 | 1 |
| Juniperus navicularis (JNAV) | 1 | 1 | 1 | 1 | 1 | 1 | 1 | 1 | NA | 1 | 1 | 1 | 1 | 1 | 1 | 1 | 1 | NA |
| Juniperus oxycedrus (JOXY) | 1 | 1 | 1 | 1 | 1 | 1 | 1 | 1 | 1 | 1 | 1 | 1 | 1 | 1 | 1 | 1 | 1 | 1 |
| Juniperus phoenicea (JPHO) | 1 | 1 | 1 | 1 | 1 | 1 | 1 | 1 | 1 | 1 | 1 | 1 | 1 | 1 | 1 | 1 | 1 | 1 |
| Juniperus thurifera (JTHU) | 1 | 1 | 1 | 1 | 1 | 1 | 1 | 1 | 1 | 1 | 1 | 1 | 1 | 1 | 1 | 1 | 1 | 1 |
| Laurus nobilis (LNOB) | 1 | 1 | 1 | 1 | 1 | 1 | 1 | 1 | 1 | 1 | 1 | 1 | 1 | 1 | 1 | 1 | 1 | 1 |
| Liquidambar orientalis (LORI) | 1 | 1 | 1 | 1 | 1 | 1 | 1 | 1 | 1 | 1 | 1 | 1 | 1 | 1 | 1 | 1 | NA | NA |
| Lonicera arborea (LARB) | 1 | 1 | 1 | 1 | 1 | 1 | 1 | 1 | 1 | 1 | 1 | 1 | 1 | 1 | 1 | 1 | NA | NA |
| Malus dasyphylla (MDAS) | 1 | 1 | 1 | 1 | 1 | 1 | 1 | 1 | 1 | 1 | 1 | 1 | 1 | 1 | NA | 1 | NA | NA |
| Malus florentina (MFLO) | 1 | 1 | 1 | 1 | 1 | 1 | 1 | 1 | 1 | 1 | 1 | 1 | 1 | 1 | 1 | 1 | 1 | 1 |
| Malus sylvestris (MSYL) | 1 | 1 | 1 | 1 | 1 | 1 | 1 | 1 | 1 | 1 | 1 | 1 | 1 | 1 | 1 | 1 | 1 | 1 |
| Malus trilobata (MTRI) | 1 | 1 | 1 | 1 | 1 | 1 | 1 | 1 | 1 | 1 | 1 | 1 | 1 | 1 | 1 | 1 | NA | NA |
| Myrica faya (MFAY) | 1 | 1 | 1 | 1 | 1 | 1 | 1 | 1 | 1 | 1 | 1 | 1 | 1 | 1 | NA | 1 | NA | 1 |
| Myrtus communis (MCOM) | 1 | 1 | 1 | 1 | 1 | 1 | 1 | 1 | 1 | 1 | 1 | 1 | 1 | 1 | 1 | 1 | 1 | 1 |
| Nerium oleander (NOLE) | 1 | 1 | 1 | 1 | 1 | 1 | 1 | 1 | 1 | 1 | 1 | 1 | 1 | 1 | 1 | 1 | 1 | 1 |
| Olea europaea (OEUR) | 1 | 1 | 1 | 1 | 1 | 1 | 1 | 1 | 1 | 1 | 1 | 1 | 1 | 1 | 1 | 1 | 1 | 1 |
| Ostrya carpinifolia (OCAR) | 1 | 1 | 1 | 1 | 1 | 1 | 1 | 1 | 1 | 1 | 1 | 1 | 1 | 1 | 1 | 1 | 1 | 1 |
| Phillyrea angustifolia (PANG) | 1 | 1 | 1 | 1 | 1 | 1 | 1 | 1 | 1 | 1 | 1 | 1 | 1 | 1 | 1 | 1 | 1 | 1 |
| Phillyrea latifolia (PLAT) | 1 | 1 | 1 | 1 | 1 | 1 | 1 | 1 | 1 | 1 | 1 | 1 | 1 | 1 | 1 | 1 | 1 | 1 |
| Phoenix theophrasti (PTHE) | 1 | 1 | 1 | 1 | 1 | 1 | 1 | 1 | 1 | 1 | 1 | 1 | 1 | 1 | 1 | 1 | NA | NA |
| Pinus brutia (PBRU) | 1 | 1 | 1 | 1 | 1 | 1 | 1 | 1 | 1 | 1 | 1 | 1 | 1 | 1 | 1 | 1 | 1 | 1 |
| Pinus halepensis (PHAL) | 1 | 1 | 1 | 1 | 1 | 1 | 1 | 1 | 1 | 1 | 1 | 1 | 1 | 1 | 1 | 1 | 1 | 1 |
| Pinus heldreichii (PHEL) | 1 | 1 | 1 | 1 | 1 | 1 | 1 | 1 | 1 | 1 | 1 | 1 | 1 | 1 | 1 | 1 | 1 | 1 |
| Pinus mugo (PMUG) | 1 | 1 | 1 | 1 | 1 | 1 | 1 | 1 | 1 | 1 | 1 | 1 | 1 | 1 | 1 | 1 | 1 | 1 |
| Pinus nigra (PINI) | 1 | 1 | 1 | 1 | 1 | 1 | 1 | 1 | 1 | 1 | 1 | 1 | 1 | 1 | 1 | 1 | 1 | 1 |
| Pinus pinaster (PPIR) | 1 | 1 | 1 | 1 | 1 | 1 | 1 | 1 | 1 | 1 | 1 | 1 | 1 | 1 | 1 | 1 | 1 | 1 |
| Pinus pinea (PPIA) | 1 | 1 | 1 | 1 | 1 | 1 | 1 | 1 | 1 | 1 | 1 | 1 | 1 | 1 | 1 | 1 | 1 | 1 |
| Pinus sylvestris (PSYL) | 1 | 1 | 1 | 1 | 1 | 1 | 1 | 1 | 1 | 1 | 1 | 1 | 1 | 1 | 1 | 1 | 1 | 1 |
| Pinus uncinata (PUNC) | 1 | 1 | 1 | 1 | 1 | 1 | 1 | 1 | 1 | 1 | 1 | 1 | 1 | 1 | 1 | 1 | 1 | 1 |
| Pistacia atlantica (PATL) | 1 | 1 | 1 | 1 | 1 | 1 | 1 | 1 | 1 | 1 | 1 | 1 | 1 | 1 | 1 | 1 | 1 | 1 |
| Pistacia lentiscus (PLEN) | 1 | 1 | 1 | 1 | 1 | 1 | 1 | 1 | 1 | 1 | 1 | 1 | 1 | 1 | 1 | 1 | 1 | 1 |
| Pistacia terebinthus (PTER) | 1 | 1 | 1 | 1 | 1 | 1 | 1 | 1 | 1 | 1 | 1 | 1 | 1 | 1 | 1 | 1 | 1 | 1 |
| Platanus orientalis (PORI) | 1 | 1 | 1 | 1 | 1 | 1 | 1 | 1 | 1 | 1 | 1 | 1 | 1 | 1 | 1 | 1 | 1 | 1 |
| Populus alba (PALB) | 1 | 1 | 1 | 1 | 1 | 1 | 1 | 1 | 1 | 1 | 1 | 1 | 1 | 1 | 1 | 1 | 1 | 1 |
| Populus nigra (PONI) | 1 | 1 | 1 | 1 | 1 | 1 | 1 | 1 | 1 | 1 | 1 | 1 | 1 | 1 | 1 | 1 | 1 | 1 |
| Populus tremula (PTRE) | 1 | 1 | 1 | 1 | 1 | 1 | 1 | 1 | 1 | 1 | 1 | 1 | 1 | 1 | 1 | 1 | 1 | 1 |
| Prunus avium (PAVI) | 1 | 1 | 1 | 1 | 1 | 1 | 1 | 1 | 1 | 1 | 1 | 1 | 1 | 1 | 1 | 1 | 1 | 1 |
| Prunus brigantina (PBRI) | 1 | 1 | 1 | 1 | 1 | 1 | 1 | 1 | 1 | 1 | 1 | 1 | 1 | 1 | 1 | 1 | 1 | 1 |
| Prunus cerasifera (PCER) | 1 | 1 | 1 | 1 | 1 | 1 | 1 | 1 | 1 | 1 | 1 | 1 | 1 | 1 | 1 | 1 | 1 | 1 |
| Prunus cocomilia (PCOC) | 1 | 1 | 1 | 1 | 1 | 1 | 1 | 1 | 1 | 1 | 1 | 1 | 1 | 1 | 1 | 1 | 1 | 1 |
| Prunus lusitanica (PLUS) | 1 | 1 | 1 | 1 | 1 | 1 | 1 | 1 | 1 | 1 | 1 | 1 | 1 | 1 | 1 | 1 | 1 | 1 |
| Prunus mahaleb (PMAH) | 1 | 1 | 1 | 1 | 1 | 1 | 1 | 1 | 1 | 1 | 1 | 1 | 1 | 1 | 1 | 1 | 1 | 1 |
| Prunus padus (PPAD) | 1 | 1 | 1 | 1 | 1 | 1 | 1 | 1 | 1 | 1 | 1 | 1 | 1 | 1 | 1 | 1 | 1 | 1 |
| Prunus webbii (PWEB) | 1 | 1 | 1 | 1 | 1 | 1 | 1 | 1 | 1 | 1 | 1 | 1 | 1 | 1 | 1 | 1 | 1 | 1 |
| Pyrus bourgaeana (PBOU) | 1 | 1 | 1 | 1 | 1 | 1 | 1 | 1 | 1 | 1 | 1 | 1 | 1 | 1 | 1 | 1 | 1 | 1 |
| Pyrus cordata (PCOR) | 1 | 1 | 1 | 1 | 1 | 1 | 1 | 1 | 1 | 1 | 1 | 1 | 1 | 1 | 1 | 1 | NA | NA |
| Pyrus elaeagrifolia (PELA) | NA | 1 | 1 | 1 | 1 | 1 | 1 | 1 | 1 | 1 | 1 | 1 | 1 | 1 | 1 | 1 | NA | NA |
| Pyrus pyraster (PPYR) | 1 | 1 | 1 | 1 | 1 | 1 | 1 | 1 | 1 | 1 | 1 | 1 | 1 | 1 | 1 | 1 | 1 | 1 |
| Pyrus spinosa (PSPI) | 1 | 1 | 1 | 1 | 1 | 1 | 1 | 1 | 1 | 1 | 1 | 1 | 1 | 1 | 1 | 1 | 1 | 1 |
| Pyrus syriaca (PSYR) | NA | 1 | 1 | 1 | 1 | 1 | 1 | 1 | 1 | 1 | 1 | 1 | 1 | 1 | 1 | 1 | NA | 1 |
| Quercus alnifolia (QALN) | 1 | 1 | 1 | 1 | 1 | 1 | 1 | 1 | 1 | 1 | 1 | 1 | 1 | 1 | 1 | 1 | NA | 1 |
| Quercus aucheri (QAUC) | 1 | NA | NA | NA | NA | 1 | 1 | 1 | 1 | 1 | 1 | 1 | 1 | 1 | NA | 1 | NA | NA |
| Quercus canariensis (QCAN) | 1 | 1 | 1 | 1 | 1 | 1 | 1 | 1 | 1 | 1 | 1 | 1 | 1 | 1 | 1 | 1 | 1 | 1 |
| Quercus cerris (QCER) | 1 | 1 | 1 | 1 | 1 | 1 | 1 | 1 | 1 | 1 | 1 | 1 | 1 | 1 | 1 | 1 | 1 | 1 |
| Quercus coccifera (QCOC) | 1 | 1 | 1 | 1 | 1 | 1 | 1 | 1 | 1 | 1 | 1 | 1 | 1 | 1 | 1 | 1 | 1 | 1 |
| Quercus congesta (QCON) | 1 | 1 | 1 | 1 | 1 | 1 | 1 | 1 | 1 | 1 | 1 | 1 | 1 | 1 | 1 | 1 | 1 | 1 |
| Quercus crenata (QCRE) | 1 | 1 | 1 | 1 | 1 | 1 | 1 | 1 | 1 | 1 | 1 | 1 | 1 | 1 | NA | 1 | 1 | 1 |
| Quercus dalechampii (QDAL) | 1 | 1 | 1 | 1 | 1 | 1 | 1 | 1 | 1 | 1 | 1 | 1 | 1 | 1 | 1 | 1 | 1 | 1 |
| Quercus faginea (QFAG) | 1 | 1 | 1 | 1 | 1 | 1 | 1 | 1 | 1 | 1 | 1 | 1 | 1 | 1 | 1 | 1 | 1 | 1 |
| Quercus frainetto (QFRA) | 1 | 1 | 1 | 1 | 1 | 1 | 1 | 1 | 1 | 1 | 1 | 1 | 1 | 1 | 1 | 1 | 1 | 1 |
| Quercus gussonei (QGUS) | 1 | 1 | 1 | 1 | 1 | 1 | 1 | 1 | 1 | 1 | 1 | 1 | 1 | 1 | 1 | 1 | 1 | 1 |
| Quercus ichnusae (QICH) | 1 | 1 | 1 | 1 | 1 | 1 | 1 | 1 | 1 | 1 | 1 | 1 | 1 | 1 | 1 | 1 | 1 | 1 |
| Quercus ilex (QILE) | 1 | 1 | 1 | 1 | 1 | 1 | 1 | 1 | 1 | 1 | 1 | 1 | 1 | 1 | 1 | 1 | 1 | 1 |
| Quercus infectoria (QINF) | 1 | 1 | 1 | 1 | 1 | 1 | 1 | 1 | 1 | 1 | 1 | 1 | 1 | 1 | NA | 1 | 1 | 1 |
| Quercus ithaburensis (QITH) | 1 | 1 | 1 | 1 | 1 | 1 | 1 | 1 | 1 | 1 | 1 | 1 | 1 | 1 | 1 | 1 | 1 | 1 |
| Quercus petraea (QPET) | 1 | 1 | 1 | 1 | 1 | 1 | 1 | 1 | 1 | 1 | 1 | 1 | 1 | 1 | 1 | 1 | 1 | 1 |
| Quercus pubescens (QPUB) | 1 | 1 | 1 | 1 | 1 | 1 | 1 | 1 | 1 | 1 | 1 | 1 | 1 | 1 | 1 | 1 | 1 | 1 |
| Quercus pyrenaica (QPYR) | 1 | 1 | 1 | 1 | 1 | 1 | 1 | 1 | 1 | 1 | 1 | 1 | 1 | 1 | 1 | 1 | 1 | 1 |
| Quercus robur (QROB) | 1 | 1 | 1 | 1 | 1 | 1 | 1 | 1 | 1 | 1 | 1 | 1 | 1 | 1 | 1 | 1 | 1 | 1 |
| Quercus suber (QSUB) | 1 | 1 | 1 | 1 | 1 | 1 | 1 | 1 | 1 | 1 | 1 | 1 | 1 | 1 | 1 | 1 | 1 | 1 |
| Quercus trojana (QTRO) | 1 | 1 | 1 | 1 | 1 | 1 | 1 | 1 | 1 | 1 | 1 | 1 | 1 | 1 | 1 | 1 | 1 | 1 |
| Rhamnus alaternus (RALA) | 1 | 1 | 1 | 1 | 1 | 1 | 1 | 1 | 1 | 1 | 1 | 1 | 1 | 1 | 1 | 1 | 1 | 1 |
| Rhamnus cathartica (RCAT) | 1 | 1 | 1 | 1 | 1 | 1 | 1 | 1 | 1 | 1 | 1 | 1 | 1 | 1 | 1 | 1 | 1 | 1 |
| Rhamnus persicifolia (RPER) | 1 | 1 | 1 | 1 | 1 | 1 | 1 | 1 | 1 | 1 | 1 | 1 | 1 | 1 | 1 | 1 | 1 | 1 |
| Rhododendron ponticum (RPON) | 1 | 1 | 1 | 1 | 1 | 1 | 1 | 1 | 1 | 1 | 1 | 1 | 1 | 1 | 1 | 1 | 1 | NA |
| Salix alba (SALB) | 1 | 1 | 1 | 1 | 1 | 1 | 1 | 1 | 1 | 1 | 1 | 1 | 1 | 1 | 1 | 1 | 1 | 1 |
| Salix amplexicaulis (SAMP) | 1 | 1 | 1 | 1 | 1 | 1 | 1 | 1 | 1 | 1 | 1 | 1 | 1 | 1 | NA | 1 | NA | NA |
| Salix apennina (SAPE) | 1 | 1 | 1 | 1 | 1 | 1 | 1 | 1 | 1 | 1 | 1 | 1 | 1 | 1 | NA | 1 | 1 | 1 |
| Salix appendiculata (SAPP) | 1 | 1 | 1 | 1 | 1 | 1 | 1 | 1 | 1 | 1 | 1 | 1 | 1 | 1 | 1 | 1 | 1 | NA |
| Salix arrigonii (SARR) | 1 | 1 | 1 | 1 | 1 | 1 | 1 | 1 | 1 | 1 | 1 | 1 | 1 | 1 | NA | 1 | 1 | 1 |
| Salix caprea (SCAP) | 1 | 1 | 1 | 1 | 1 | 1 | 1 | 1 | 1 | 1 | 1 | 1 | 1 | 1 | 1 | 1 | 1 | 1 |
| Salix cinerea (SCIN) | 1 | 1 | 1 | 1 | 1 | 1 | 1 | 1 | 1 | 1 | 1 | 1 | 1 | 1 | 1 | 1 | 1 | 1 |
| Salix eleagnos (SELE) | 1 | 1 | 1 | 1 | 1 | 1 | 1 | 1 | 1 | 1 | 1 | 1 | 1 | 1 | 1 | 1 | 1 | 1 |
| Salix fragilis (SFRA) | 1 | 1 | 1 | 1 | 1 | 1 | 1 | 1 | 1 | 1 | 1 | 1 | 1 | 1 | 1 | 1 | 1 | 1 |
| Salix gussonei (SGUS) | 1 | 1 | 1 | 1 | 1 | 1 | 1 | 1 | 1 | 1 | 1 | 1 | 1 | 1 | NA | 1 | 1 | 1 |
| Salix pedicellata (SPED) | 1 | 1 | 1 | 1 | 1 | 1 | 1 | 1 | 1 | 1 | 1 | 1 | 1 | 1 | NA | 1 | 1 | 1 |
| Salix pentandra (SPEN) | 1 | 1 | 1 | 1 | 1 | 1 | 1 | 1 | 1 | 1 | 1 | 1 | 1 | 1 | 1 | 1 | 1 | NA |
| Salix purpurea (SPUR) | 1 | 1 | 1 | 1 | 1 | 1 | 1 | 1 | 1 | 1 | 1 | 1 | 1 | 1 | 1 | 1 | 1 | 1 |
| Salix salviifolia (SSAL) | 1 | 1 | 1 | 1 | 1 | 1 | 1 | 1 | 1 | 1 | 1 | 1 | 1 | 1 | NA | 1 | NA | NA |
| Salix triandra (SATR) | 1 | 1 | 1 | 1 | 1 | 1 | 1 | 1 | 1 | 1 | 1 | 1 | 1 | 1 | 1 | 1 | 1 | 1 |
| Salix viminalis (SVIM) | 1 | 1 | 1 | 1 | 1 | 1 | 1 | 1 | 1 | 1 | 1 | 1 | 1 | 1 | NA | 1 | 1 | NA |
| Salix xanthicola (SXAN) | 1 | 1 | 1 | 1 | 1 | 1 | 1 | 1 | 1 | 1 | 1 | 1 | 1 | 1 | NA | 1 | NA | NA |
| Sambucus nigra (SNIG) | 1 | 1 | 1 | 1 | 1 | 1 | 1 | 1 | 1 | 1 | 1 | 1 | 1 | 1 | 1 | 1 | 1 | 1 |
| Sambucus racemosa (SRAC) | 1 | 1 | 1 | 1 | 1 | 1 | 1 | 1 | 1 | 1 | 1 | 1 | 1 | 1 | 1 | 1 | 1 | 1 |
| Searsia tripartita (SETR) | 1 | 1 | 1 | 1 | 1 | 1 | 1 | 1 | 1 | 1 | 1 | 1 | 1 | 1 | NA | 1 | 1 | 1 |
| Sorbus aria (SARI) | 1 | 1 | 1 | 1 | 1 | 1 | 1 | 1 | 1 | 1 | 1 | 1 | 1 | 1 | 1 | 1 | 1 | 1 |
| Sorbus aucuparia (SAUC) | 1 | 1 | 1 | 1 | 1 | 1 | 1 | 1 | 1 | 1 | 1 | 1 | 1 | 1 | 1 | 1 | 1 | 1 |
| Sorbus austriaca (SAUS) | 1 | 1 | 1 | 1 | 1 | 1 | 1 | 1 | NA | 1 | 1 | 1 | 1 | 1 | NA | 1 | NA | NA |
| Sorbus domestica (SDOM) | 1 | 1 | 1 | 1 | 1 | 1 | 1 | 1 | 1 | 1 | 1 | 1 | 1 | 1 | 1 | 1 | 1 | 1 |
| Sorbus graeca (SGRA) | 1 | 1 | 1 | 1 | 1 | 1 | 1 | 1 | 1 | 1 | 1 | 1 | 1 | 1 | 1 | 1 | 1 | 1 |
| Sorbus latifolia (SLAT) | 1 | 1 | 1 | 1 | 1 | 1 | 1 | 1 | 1 | 1 | 1 | 1 | 1 | 1 | 1 | 1 | NA | NA |
| Sorbus mougeotii (SMOU) | 1 | 1 | 1 | 1 | 1 | 1 | 1 | 1 | 1 | 1 | 1 | 1 | 1 | 1 | 1 | 1 | 1 | 1 |
| Sorbus torminalis (STOR) | 1 | 1 | 1 | 1 | 1 | 1 | 1 | 1 | 1 | 1 | 1 | 1 | 1 | 1 | 1 | 1 | 1 | 1 |
| Sorbus umbellata (SUMB) | 1 | 1 | 1 | 1 | 1 | 1 | 1 | 1 | 1 | 1 | 1 | 1 | 1 | 1 | 1 | 1 | NA | NA |
| Spartium junceum (SJUN) | 1 | 1 | 1 | 1 | 1 | 1 | 1 | 1 | 1 | 1 | 1 | 1 | 1 | 1 | 1 | 1 | 1 | 1 |
| Staphylea pinnata (SPIN) | 1 | 1 | 1 | 1 | 1 | 1 | 1 | 1 | 1 | 1 | 1 | 1 | 1 | 1 | 1 | 1 | 1 | 1 |
| Styrax officinalis (SOFF) | 1 | 1 | 1 | 1 | 1 | 1 | 1 | 1 | 1 | 1 | 1 | 1 | 1 | 1 | 1 | 1 | 1 | 1 |
| Tamarix africana (TAFR) | 1 | 1 | 1 | 1 | 1 | 1 | 1 | 1 | NA | 1 | 1 | 1 | 1 | 1 | 1 | 1 | 1 | 1 |
| Tamarix arborea (TARB) | 1 | 1 | 1 | 1 | 1 | 1 | 1 | 1 | NA | 1 | 1 | 1 | 1 | 1 | NA | 1 | 1 | 1 |
| Tamarix boveana (TBOV) | 1 | 1 | 1 | 1 | 1 | 1 | 1 | 1 | NA | 1 | 1 | 1 | 1 | 1 | NA | 1 | NA | 1 |
| Tamarix canariensis (TCAN) | 1 | 1 | 1 | 1 | 1 | 1 | 1 | 1 | 1 | 1 | 1 | 1 | 1 | 1 | NA | 1 | 1 | 1 |
| Tamarix dalmatica (TDAL) | 1 | 1 | 1 | 1 | 1 | 1 | 1 | 1 | NA | 1 | 1 | 1 | 1 | 1 | NA | 1 | NA | 1 |
| Tamarix gallica (TGAL) | 1 | 1 | 1 | 1 | 1 | 1 | 1 | 1 | NA | 1 | 1 | 1 | 1 | 1 | 1 | 1 | 1 | 1 |
| Tamarix hampeana (THAM) | 1 | 1 | 1 | 1 | 1 | 1 | 1 | 1 | NA | 1 | 1 | 1 | 1 | 1 | NA | 1 | 1 | 1 |
| Tamarix laxa (TLAX) | 1 | 1 | 1 | 1 | 1 | 1 | 1 | 1 | NA | 1 | 1 | 1 | 1 | 1 | NA | 1 | NA | NA |
| Tamarix mascatensis (TMAS) | 1 | 1 | 1 | 1 | 1 | 1 | 1 | 1 | NA | 1 | 1 | 1 | 1 | 1 | NA | 1 | NA | NA |
| Tamarix minoa (TMIN) | 1 | 1 | 1 | 1 | 1 | 1 | 1 | 1 | NA | 1 | 1 | 1 | 1 | 1 | NA | 1 | NA | NA |
| Tamarix parviflora (TPAR) | 1 | 1 | 1 | 1 | 1 | 1 | 1 | 1 | NA | 1 | 1 | 1 | 1 | 1 | NA | 1 | 1 | 1 |
| Tamarix passerinoides (TPAS) | 1 | 1 | 1 | 1 | 1 | 1 | 1 | 1 | NA | 1 | 1 | 1 | 1 | 1 | NA | 1 | NA | NA |
| Tamarix ramosissima (TRAM) | 1 | 1 | 1 | 1 | 1 | 1 | 1 | 1 | NA | 1 | 1 | 1 | 1 | 1 | 1 | 1 | 1 | 1 |
| Tamarix smyrnensis (TSMY) | 1 | 1 | 1 | 1 | 1 | 1 | 1 | 1 | 1 | 1 | 1 | 1 | 1 | 1 | NA | 1 | NA | 1 |
| Tamarix tetragyna (TTEG) | 1 | 1 | 1 | 1 | 1 | 1 | 1 | 1 | NA | 1 | 1 | 1 | 1 | 1 | NA | 1 | 1 | 1 |
| Tamarix tetrandra (TTEN) | 1 | 1 | 1 | 1 | 1 | 1 | 1 | 1 | 1 | 1 | 1 | 1 | 1 | 1 | 1 | 1 | NA | 1 |
| Taxus baccata (TBAC) | 1 | 1 | 1 | 1 | 1 | 1 | 1 | 1 | 1 | 1 | 1 | 1 | 1 | 1 | 1 | 1 | 1 | 1 |
| Tetraclinis articulata (TART) | 1 | 1 | 1 | 1 | 1 | 1 | 1 | 1 | 1 | 1 | 1 | 1 | 1 | 1 | 1 | 1 | 1 | 1 |
| Tilia cordata (TCOR) | 1 | 1 | 1 | 1 | 1 | 1 | 1 | 1 | 1 | 1 | 1 | 1 | 1 | 1 | 1 | 1 | 1 | 1 |
| Tilia platyphyllos (TPLA) | 1 | 1 | 1 | 1 | 1 | 1 | 1 | 1 | 1 | 1 | 1 | 1 | 1 | 1 | 1 | 1 | 1 | 1 |
| Tilia tomentosa (TTOM) | 1 | 1 | 1 | 1 | 1 | 1 | 1 | 1 | 1 | 1 | 1 | 1 | 1 | 1 | 1 | 1 | NA | NA |
| Ulmus canescens (UCAN) | 1 | 1 | 1 | 1 | 1 | 1 | 1 | 1 | 1 | 1 | 1 | 1 | 1 | 1 | NA | 1 | 1 | 1 |
| Ulmus glabra (UGLA) | 1 | 1 | 1 | 1 | 1 | 1 | 1 | 1 | 1 | 1 | 1 | 1 | 1 | 1 | 1 | 1 | 1 | 1 |
| Ulmus laevis (ULAE) | 1 | 1 | 1 | 1 | 1 | 1 | 1 | 1 | 1 | 1 | 1 | 1 | 1 | 1 | 1 | 1 | 1 | 1 |
| Ulmus minor (UMIN) | 1 | 1 | 1 | 1 | 1 | 1 | 1 | 1 | 1 | 1 | 1 | 1 | 1 | 1 | 1 | 1 | 1 | 1 |
| Ulmus procera (UPRO) | 1 | 1 | 1 | 1 | 1 | 1 | 1 | 1 | 1 | 1 | 1 | 1 | 1 | 1 | NA | 1 | NA | 1 |
| Vitex agnus-castus (VAGN) | 1 | 1 | 1 | 1 | 1 | 1 | 1 | 1 | 1 | 1 | 1 | 1 | 1 | 1 | 1 | 1 | 1 | 1 |
| Zelkova abelicea (ZABE) | 1 | 1 | 1 | 1 | 1 | 1 | 1 | 1 | 1 | 1 | 1 | 1 | 1 | 1 | NA | 1 | NA | NA |
| Zelkova sicula (ZSIC) | 1 | 1 | 1 | 1 | 1 | 1 | 1 | 1 | 1 | 1 | 1 | 1 | 1 | 1 | NA | 1 | 1 | NA |
| TOTAL number of species with available data | 207 | 207 | 207 | 207 | 207 | 210 | 210 | 209 | 186 | 210 | 210 | 207 | 210 | 208 | 172 | 210 | 159 | 166 |

***Supplementary Table 4. Source of the topological placement of each of the 210 considered tree species in WOODIV data base.*** *“GBOTB.extended” refers to Smith and Brown, 2018, Jin and Qian, 2019 mega-phylogeny, and “WOODIV_v1” refers to the phylogenetic tree in WOODIV version 1.*

| **Species (spcode)** | **Topology source** |
| --- | --- |
| Abies alba (AALB) | GBOTB.extended |
| Abies borisii-regis (ABOR) | WOODIV_v1 |
| Abies cephalonica (ACEP) | GBOTB.extended |
| Abies nebrodensis (ANEB) | GBOTB.extended |
| Abies pinsapo (APIN) | GBOTB.extended |
| Acer campestre (ACAM) | GBOTB.extended |
| Acer cappadocicum (ACAP) | GBOTB.extended |
| Acer heldreichii (AHEL) | GBOTB.extended |
| Acer hyrcanum (AHYR) | GBOTB.extended |
| Acer monspessulanum (AMON) | GBOTB.extended |
| Acer obtusifolium (AOBT) | GBOTB.extended |
| Acer opalus (AOPA) | GBOTB.extended |
| Acer platanoides (APLA) | GBOTB.extended |
| Acer pseudoplatanus (APSE) | GBOTB.extended |
| Acer sempervirens (ASEM) | GBOTB.extended |
| Acer tataricum (ATAT) | GBOTB.extended |
| Aesculus hippocastanum (AHIP) | GBOTB.extended |
| Alnus cordata (ACOR) | GBOTB.extended |
| Alnus glutinosa (AGLU) | GBOTB.extended |
| Alnus incana (AINC) | GBOTB.extended |
| Alnus lusitanica (ALUS) | Vit et al., 2017. Two new polyploid species closely related to Alnus glutinosa in Europe and North Africa – An analysis based on morphometry, karyology, flow cytometry and microsatellites. Taxon 66, 567-583 [taxonomic proximity] |
| Alnus orientalis (AORI) | GBOTB.extended |
| Alnus rohlenae (AROH) | WOODIV_v1 |
| Alnus viridis (AVIR) | WOODIV_v1 |
| Arbutus andrachne (AAND) | GBOTB.extended |
| Arbutus unedo (AUNE) | GBOTB.extended |
| Betula pendula (BPEN) | GBOTB.extended |
| Betula pubescens (BPUB) | GBOTB.extended |
| Buxus balearica (BBAL) | GBOTB.extended |
| Buxus sempervirens (BSEM) | GBOTB.extended |
| Carpinus betulus (CBET) | GBOTB.extended |
| Carpinus orientalis (CAOR) | GBOTB.extended |
| Castanea sativa (CSAT) | GBOTB.extended |
| Cedrus libani (CLIB) | GBOTB.extended |
| Celtis australis (CAUS) | WOODIV_v1 |
| Celtis planchoniana (CPLA) | WOODIV_v1 |
| Celtis tournefortii (CTOU) | WOODIV_v1 |
| Ceratonia siliqua (CSIA) | GBOTB.extended |
| Cercis siliquastrum (CSIM) | GBOTB.extended |
| Chamaerops humilis (CHUM) | GBOTB.extended |
| Cotinus coggygria (CCOG) | GBOTB.extended |
| Corylus avellana (CAVE) | GBOTB.extended |
| Corylus colurna (CCOL) | GBOTB.extended |
| Cotoneaster granatensis (CGRA) | WOODIV_v1 |
| Crataegus azarolus (CAZA) | GBOTB.extended |
| Crataegus heldreichii (CHEL) | Lo and Donoghue, 2012. Expanded phylogenetic and dating analyses of the apples and their relatives (Pyreae, Rosaceae). Mol Phylogenet Evol 63, 230-243 |
| Crataegus laciniata (CLAC) | WOODIV_v1 |
| Crataegus laevigata (CLAE) | GBOTB.extended |
| Crataegus monogyna (CMON) | GBOTB.extended |
| Crataegus nevadensis (CNEV) | WOODIV_v1 |
| Crataegus orientalis (CROR) | WOODIV_v1 |
| Crataegus pentagyna (CPEN) | GBOTB.extended |
| Crataegus pycnoloba (CPYC) | WOODIV_v1 |
| Cupressus sempervirens (CSEM) | GBOTB.extended |
| Cytisus aeolicus (CAEO) | Cheikh Albassatneh et al., 2020. A comprehensive, genus-level time-calibrated phylogeny of the tree flora of Mediterranean Europe and an assessment of its vulnerability. Botany Letters 167, 276-289 |
| Erica arborea (EARB) | GBOTB.extended |
| Fagus orientalis (FORI) | WOODIV_v1 |
| Fagus sylvatica (FSYL) | WOODIV_v1 |
| Ficus carica (FCAR) | GBOTB.extended |
| Fontanesia philliraeoides (FPHI) | GBOTB.extended |
| Frangula alnus (FALN) | GBOTB.extended |
| Fraxinus angustifolia (FANG) | GBOTB.extended |
| Fraxinus excelsior (FEXC) | GBOTB.extended |
| Fraxinus ornus (FORN) | GBOTB.extended |
| Fraxinus pallisae (FPAL) | WOODIV_v1 |
| Genista etnensis (GETN) | Cheikh Albassatneh et al., 2020. A comprehensive, genus-level time-calibrated phylogeny of the tree flora of Mediterranean Europe and an assessment of its vulnerability. Botany Letters 167, 276-289 |
| Genista tyrrhena (GTYR) | Cheikh Albassatneh et al., 2020. A comprehensive, genus-level time-calibrated phylogeny of the tree flora of Mediterranean Europe and an assessment of its vulnerability. Botany Letters 167, 276-289 |
| Ilex aquifolium (IAQU) | GBOTB.extended |
| Juglans regia (JREG) | GBOTB.extended |
| Juniperus communis (JCOM) | GBOTB.extended |
| Juniperus deltoides (JDEL) | GBOTB.extended |
| Juniperus drupacea (JDRU) | GBOTB.extended |
| Juniperus excelsa (JEXC) | GBOTB.extended |
| Juniperus foetidissima (JFOE) | Güvendiren, 2015. Molecular phylogenetic analyses of Juniperus L. species in Turkey and their relations with other Junipers based on cpDNA. PhD thesis, Middle East Technical University |
| Juniperus macrocarpa (JMAC) | GBOTB.extended |
| Juniperus navicularis (JNAV) | GBOTB.extended |
| Juniperus oxycedrus (JOXY) | GBOTB.extended |
| Juniperus phoenicea (JPHO) | GBOTB.extended |
| Juniperus thurifera (JTHU) | GBOTB.extended |
| Laurus nobilis (LNOB) | Cheikh Albassatneh et al., 2020. A comprehensive, genus-level time-calibrated phylogeny of the tree flora of Mediterranean Europe and an assessment of its vulnerability. Botany Letters 167, 276-289 |
| Liquidambar orientalis (LORI) | GBOTB.extended |
| Lonicera arborea (LARB) | Cheikh Albassatneh et al., 2020. A comprehensive, genus-level time-calibrated phylogeny of the tree flora of Mediterranean Europe and an assessment of its vulnerability. Botany Letters 167, 276-289 |
| Malus dasyphylla (MDAS) | WOODIV_v1 |
| Malus florentina (MFLO) | Wang et al., 2019. The complete chloroplast genome sequence of Docynia indica (Wall.) Decne. Mitochondr DNA Part B 4, 3046-3048 |
| Malus sylvestris (MSYL) | GBOTB.extended |
| Malus trilobata (MTRI) | GBOTB.extended |
| Myrica faya (MFAY) | WOODIV_v1 |
| Myrtus communis (MCOM) | GBOTB.extended |
| Nerium oleander (NOLE) | GBOTB.extended |
| Olea europaea (OEUR) | GBOTB.extended |
| Ostrya carpinifolia (OCAR) | GBOTB.extended |
| Phillyrea angustifolia (PANG) | GBOTB.extended |
| Phillyrea latifolia (PLAT) | GBOTB.extended |
| Phoenix theophrasti (PTHE) | GBOTB.extended |
| Pinus brutia (PBRU) | GBOTB.extended |
| Pinus halepensis (PHAL) | GBOTB.extended |
| Pinus heldreichii (PHEL) | WOODIV_v1 |
| Pinus mugo (PMUG) | GBOTB.extended |
| Pinus nigra (PINI) | GBOTB.extended |
| Pinus pinaster (PPIR) | GBOTB.extended |
| Pinus pinea (PPIA) | GBOTB.extended |
| Pinus sylvestris (PSYL) | GBOTB.extended |
| Pinus uncinata (PUNC) | GBOTB.extended |
| Pistacia atlantica (PATL) | GBOTB.extended |
| Pistacia lentiscus (PLEN) | GBOTB.extended |
| Pistacia terebinthus (PTER) | GBOTB.extended |
| Platanus orientalis (PORI) | GBOTB.extended |
| Populus alba (PALB) | GBOTB.extended |
| Populus nigra (PONI) | GBOTB.extended |
| Populus tremula (PTRE) | GBOTB.extended |
| Prunus avium (PAVI) | GBOTB.extended |
| Prunus brigantina (PBRI) | GBOTB.extended |
| Prunus cerasifera (PCER) | GBOTB.extended |
| Prunus cocomilia (PCOC) | GBOTB.extended |
| Prunus lusitanica (PLUS) | GBOTB.extended |
| Prunus mahaleb (PMAH) | GBOTB.extended |
| Prunus padus (PPAD) | GBOTB.extended |
| Prunus webbii (PWEB) | WOODIV_v1 |
| Pyrus bourgaeana (PBOU) | WOODIV_v1 |
| Pyrus cordata (PCOR) | GBOTB.extended |
| Pyrus elaeagrifolia (PELA) | GBOTB.extended |
| Pyrus pyraster (PPYR) | GBOTB.extended |
| Pyrus spinosa (PSPI) | GBOTB.extended |
| Pyrus syriaca (PSYR) | GBOTB.extended |
| Quercus alnifolia (QALN) | GBOTB.extended |
| Quercus aucheri (QAUC) | GBOTB.extended |
| Quercus canariensis (QCAN) | GBOTB.extended |
| Quercus cerris (QCER) | GBOTB.extended |
| Quercus coccifera (QCOC) | GBOTB.extended |
| Quercus congesta (QCON) | WOODIV_v1 |
| Quercus crenata (QCRE) | GBOTB.extended |
| Quercus dalechampii (QDAL) | GBOTB.extended |
| Quercus faginea (QFAG) | GBOTB.extended |
| Quercus frainetto (QFRA) | GBOTB.extended |
| Quercus gussonei (QGUS) | WOODIV_v1 |
| Quercus ichnusae (QICH) | WOODIV_v1 |
| Quercus ilex (QILE) | Hipp et al., 2020. Genomic landscape of the global oak phylogeny. New Phytol 226, 1198-1212 |
| Quercus infectoria (QINF) | Hipp et al., 2020. Genomic landscape of the global oak phylogeny. New Phytol 226, 1198-1212 |
| Quercus ithaburensis (QITH) | Hipp et al., 2020. Genomic landscape of the global oak phylogeny. New Phytol 226, 1198-1212 |
| Quercus petraea (QPET) | GBOTB.extended |
| Quercus pubescens (QPUB) | Hipp et al., 2020. Genomic landscape of the global oak phylogeny. New Phytol 226, 1198-1212 |
| Quercus pyrenaica (QPYR) | GBOTB.extended |
| Quercus robur (QROB) | Hipp et al., 2020. Genomic landscape of the global oak phylogeny. New Phytol 226, 1198-1212 |
| Quercus suber (QSUB) | GBOTB.extended |
| Quercus trojana (QTRO) | GBOTB.extended |
| Rhamnus alaternus (RALA) | GBOTB.extended |
| Rhamnus cathartica (RCAT) | GBOTB.extended |
| Rhamnus persicifolia (RPER) | WOODIV_v1 |
| Rhododendron ponticum (RPON) | GBOTB.extended |
| Salix alba (SALB) | GBOTB.extended |
| Salix amplexicaulis (SAMP) | WOODIV_v1 |
| Salix apennina (SAPE) | WOODIV_v1 |
| Salix appendiculata (SAPP) | Wagner et al., 2021. The Evolutionary History Diversity and Ecology of Willows (Salix L.) in the European Alps. Diversity 13, 146 |
| Salix arrigonii (SARR) | WOODIV_v1 |
| Salix caprea (SCAP) | GBOTB.extended |
| Salix cinerea (SCIN) | GBOTB.extended |
| Salix eleagnos (SELE) | GBOTB.extended |
| Salix fragilis (SFRA) | WOODIV_v1 |
| Salix gussonei (SGUS) | WOODIV_v1 |
| Salix pedicellata (SPED) | GBOTB.extended |
| Salix pentandra (SPEN) | GBOTB.extended |
| Salix purpurea (SPUR) | GBOTB.extended |
| Salix salviifolia (SSAL) | Wagner et al., 2021. The Evolutionary History Diversity and Ecology of Willows (Salix L.) in the European Alps. Diversity 13, 146 |
| Salix triandra (SATR) | GBOTB.extended |
| Salix viminalis (SVIM) | GBOTB.extended |
| Salix xanthicola (SXAN) | WOODIV_v1 |
| Sambucus nigra (SNIG) | GBOTB.extended |
| Sambucus racemosa (SRAC) | GBOTB.extended |
| Searsia tripartita (SETR) | WOODIV_v1 |
| Sorbus aria (SARI) | GBOTB.extended |
| Sorbus aucuparia (SAUC) | Li et al., 2017. Molecular phylogenetics and historical biogeography of Sorbus sensu stricto (Rosaceae). Mol Phylogenet Evol 111, 76-86 |
| Sorbus austriaca (SAUS) | GBOTB.extended |
| Sorbus domestica (SDOM) | GBOTB.extended |
| Sorbus graeca (SGRA) | WOODIV_v1 |
| Sorbus latifolia (SLAT) | WOODIV_v1 |
| Sorbus mougeotii (SMOU) | WOODIV_v1 |
| Sorbus torminalis (STOR) | GBOTB.extended |
| Sorbus umbellata (SUMB) | WOODIV_v1 |
| Spartium junceum (SJUN) | GBOTB.extended |
| Staphylea pinnata (SPIN) | GBOTB.extended |
| Styrax officinalis (SOFF) | GBOTB.extended |
| Tamarix africana (TAFR) | GBOTB.extended |
| Tamarix arborea (TARB) | Villar et al., 2019. Out of the Middle East: New phylogenetic insights in the genus Tamarix (Tamaricaceae). J Syst Evol 57, 488-507 |
| Tamarix boveana (TBOV) | Villar et al., 2019. Out of the Middle East: New phylogenetic insights in the genus Tamarix (Tamaricaceae). J Syst Evol 57, 488-507 |
| Tamarix canariensis (TCAN) | Villar et al., 2019. Out of the Middle East: New phylogenetic insights in the genus Tamarix (Tamaricaceae). J Syst Evol 57, 488-507 |
| Tamarix dalmatica (TDAL) | Villar et al., 2019. Out of the Middle East: New phylogenetic insights in the genus Tamarix (Tamaricaceae). J Syst Evol 57, 488-507 |
| Tamarix gallica (TGAL) | GBOTB.extended |
| Tamarix hampeana (THAM) | Villar et al., 2019. Out of the Middle East: New phylogenetic insights in the genus Tamarix (Tamaricaceae). J Syst Evol 57, 488-507 |
| Tamarix laxa (TLAX) | GBOTB.extended |
| Tamarix mascatensis (TMAS) | WOODIV_v1 |
| Tamarix minoa (TMIN) | Villar et al., 2019. Out of the Middle East: New phylogenetic insights in the genus Tamarix (Tamaricaceae). J Syst Evol 57, 488-507 |
| Tamarix parviflora (TPAR) | GBOTB.extended |
| Tamarix passerinoides (TPAS) | GBOTB.extended |
| Tamarix ramosissima (TRAM) | GBOTB.extended |
| Tamarix smyrnensis (TSMY) | GBOTB.extended |
| Tamarix tetragyna (TTEG) | Villar et al., 2019. Out of the Middle East: New phylogenetic insights in the genus Tamarix (Tamaricaceae). J Syst Evol 57, 488-507 |
| Tamarix tetrandra (TTEN) | WOODIV_v1 |
| Taxus baccata (TBAC) | GBOTB.extended |
| Tetraclinis articulata (TART) | GBOTB.extended |
| Tilia cordata (TCOR) | GBOTB.extended |
| Tilia platyphyllos (TPLA) | GBOTB.extended |
| Tilia tomentosa (TTOM) | GBOTB.extended |
| Ulmus canescens (UCAN) | WOODIV_v1 |
| Ulmus glabra (UGLA) | GBOTB.extended |
| Ulmus laevis (ULAE) | GBOTB.extended |
| Ulmus minor (UMIN) | GBOTB.extended |
| Ulmus procera (UPRO) | WOODIV_v1 |
| Vitex agnus-castus (VAGN) | GBOTB.extended |
| Zelkova abelicea (ZABE) | GBOTB.extended |
| Zelkova sicula (ZSIC) | GBOTB.extended |

***Supplementary Table 5. Sources of the calibration points used to calibrate the WOODIV v2 phylogeny (SN = stem node, CN = crown node)****. Minimum and maximum ages are in Myr.*

| **Node** | **Age min** | **Age max** | **Source** |
| --- | --- | --- | --- |
| SN Angiosperms / Gymnosperms | 340 | 422 | Barba-Montoya et al., 2018. Constraining uncertainty in the timescale of angiosperm evolution and the veracity of a Cretaceous Terrestrial Revolution. New Phytol 218, 819-834 |
| CN Angiosperms | 138 | 210 | Barba-Montoya et al., 2018. Constraining uncertainty in the timescale of angiosperm evolution and the veracity of a Cretaceous Terrestrial Revolution. New Phytol 218, 819-834 |
| CN Eudicots | 129 | 188 | Barba-Montoya et al., 2018. Constraining uncertainty in the timescale of angiosperm evolution and the veracity of a Cretaceous Terrestrial Revolution. New Phytol 218, 819-834 |
| CN Pineacea | 206 | 312 | Ran et al., 2018. Phylogeny and evolutionary history of Pinaceae updated by transcriptomic analysis. Mol Phylogenet Evol 129, 106-116 |
| CN Gymnosperms | 240 | 325 | Ran et al., 2018. Phylogenomics resolves the deep phylogeny of seed plants and indicates partial convergent or homoplastic evolution between Gnetales and angiosperms. P Roy Soc B - Biol Sci 285, 20181012 |
| SN *Abies* / *Cedrus* | 117 | 232 | Ran et al., 2018. Phylogeny and evolutionary history of Pinaceae updated by transcriptomic analysis. Mol Phylogenet Evol 129, 106-116 |
| CN *Pinus* | 40 | 65 | Saladin et al., 2017. Fossils matter: improved estimates of divergence times in Pinus reveal older diversification. BMC Ecol Evol 17, 95 |
| CN *Abies* | 23 | 34 | Balao et al., 2020. Early diversification and permeable species boundaries in the Mediterranean firs. Ann Bot 125, 495-507 |
| SN node Malvids | 118 | 162 | Barba-Montoya et al., 2018. Constraining uncertainty in the timescale of angiosperm evolution and the veracity of a Cretaceous Terrestrial Revolution. New Phytol 218, 819-834 |
| CN Malvids | 102 | 112 | Kumar et al., 2022. TimeTree 5: An Expanded Resource for Species Divergence Times. Mol Biol Evol 39, msac174; TimeTree, https://timetree.org/, accessed on 23/06/2021 |
| CN Fabales | 48 | 69 | Kumar et al., 2022. TimeTree 5: An Expanded Resource for Species Divergence Times. Mol Biol Evol 39, msac174; TimeTree, https://timetree.org/, accessed on 23/06/2021 |
| CN Fagales | 85 | 108 | Barba-Montoya et al., 2018. Constraining uncertainty in the timescale of angiosperm evolution and the veracity of a Cretaceous Terrestrial Revolution. New Phytol 218, 819-834 |
| CN *Quercus* subgen. *Cerris* | 56 | 56 | Hipp et al., 2020. Genomic landscape of the global oak phylogeny. New Phytol 226, 1198-1212 |
| CN Monocots | 38 | 50 | Kumar et al., 2022. TimeTree 5: An Expanded Resource for Species Divergence Times. Mol Biol Evol 39, msac174; TimeTree, https://timetree.org/, accessed on 23/06/2021 |
| CN Dipsacales | 67 | 100 | Barba-Montoya et al., 2018. Constraining uncertainty in the timescale of angiosperm evolution and the veracity of a Cretaceous Terrestrial Revolution. New Phytol 218, 819-834 |
| SN *Cotoneaster* | 30 | 59 | Lo and Donoghue, 2012. Expanded phylogenetic and dating analyses of the apples and their relatives (Pyreae, Rosaceae). Mol Phylogenet Evol 63, 230-243 |
| CN Rosaceae | 84 | 100 | Lo and Donoghue, 2012. Expanded phylogenetic and dating analyses of the apples and their relatives (Pyreae, Rosaceae). Mol Phylogenet Evol 63, 230-243 |
| SN *Crataegus* | 51 | 79 | Lo and Donoghue, 2012. Expanded phylogenetic and dating analyses of the apples and their relatives (Pyreae, Rosaceae). Mol Phylogenet Evol 63, 230-243 |
| CN Rosales | 81 | 122 | Barba-Montoya et al., 2018. Constraining uncertainty in the timescale of angiosperm evolution and the veracity of a Cretaceous Terrestrial Revolution. New Phytol 218, 819-834 |
| CN *Juniperus* | 34 | 53 | Mao et al., 2010. Diversification and biogeography of Juniperus (Cupressaceae): variable diversification rates and multiple intercontinental dispersals. New Phytol 188, 254-272 |
| CN Cupressales | 190 | 220 | Condamine et al., 2020. The rise of angiosperms pushed conifers to decline during global cooling. P Natl Acad Sci USA 117, 28867-28875 |
| CN Cupressaceae | 46 | 75 | Condamine et al., 2020. The rise of angiosperms pushed conifers to decline during global cooling. P Natl Acad Sci USA 117, 28867-28875 |
| CN Ericaceae | 39 | 99 | Kumar et al., 2022. TimeTree 5: An Expanded Resource for Species Divergence Times. Mol Biol Evol 39, msac174; TimeTree, https://timetree.org/, accessed on 23/06/2021 |
| CN Superasterids | 99 | 114 | Kumar et al., 2022. TimeTree 5: An Expanded Resource for Species Divergence Times. Mol Biol Evol 39, msac174; TimeTree, https://timetree.org/, accessed on 23/06/2021 |
| CN *Acer* | 15 | 30 | Gao et al., 2020. Historical biogeography of Acer L. (Sapindaceae): genetic evidence for Out-of-Asia hypothesis with multiple dispersals to North America and Europe. Sci Rep 10, 21178. |
| CN Sapindaceae | 49 | 83 | Kumar et al., 2022. TimeTree 5: An Expanded Resource for Species Divergence Times. Mol Biol Evol 39, msac174; TimeTree, https://timetree.org/, accessed on 23/06/2021 |
| CN *Salix* | 37 | 43 | Wu et al., 2015. Phylogeny of Salix subgenus Salix s.l. (Salicaceae): delimitation, biogeography, and reticulate evolution. BMC Ecol Evol 15, 31 |
| CN Rhamnaceae | 23 | 43 | Onstein et al., 2015. Do Mediterranean-type ecosystems have a common history? - Insights from the Buckthorn family (Rhamnaceae). Evolution 69, 756-771 |
| CN *Rhamnus* | 20 | 20 | Onstein et al., 2015. Do Mediterranean-type ecosystems have a common history? - Insights from the Buckthorn family (Rhamnaceae). Evolution 69, 756-771 |
| CN Anacardiaceae | 19 | 40 | Xie et al., 2014. Biogeographic history of Pistacia (Anacardiaceae), emphasizing the evolution of the Madrean-Tethyan and the eastern Asian-Tethyan disjunctions. Mol Phylogenet Evol 77, 136-146 |
| CN *Pistacia* | 9 | 16 | Xie et al., 2014. Biogeographic history of Pistacia (Anacardiaceae), emphasizing the evolution of the Madrean-Tethyan and the eastern Asian-Tethyan disjunctions. Mol Phylogenet Evol 77, 136-146 |
| SN Core Eudicots | 150 | 175 | Barba-Montoya et al., 2018. Constraining uncertainty in the timescale of angiosperm evolution and the veracity of a Cretaceous Terrestrial Revolution. New Phytol 218, 819-834 |

***Supplementary Table 6. Description of the data included in the WOODIV v2 database*** *and the name of the corresponding file(s).*

|  | **Data name** | **Data description** | **Data file name** |
| --- | --- | --- | --- |
| **SPECIES** | Species_code | This table matches the taxon indexation code to the full name of species, and indicates the taxonomic level of the taxon (species or subspecies) and the indexation codes of the taxon when considering aggregation at species (spagg) or group (gragg) level | WOODIV_v2_Species_code.csv  [UTF-8 encoded] |
|  | Nomenclature | This table includes the taxonomic assignment of each species from the class to the subspecies level, and if the species has synonyms in Euro+Med PlantBase, Browicz or Kew databases. | WOODIV_v2_Nomenclature.csv  [UTF-8 encoded] |
|  | Endemism | This table indicates the endemism status of each species | WOODIV_v2_Endemism.csv  [UTF-8 encoded] |
| **OCCURRENCE** | Occurrence_data | This table includes the occurrences of species in the LAEA grid, the source from where the data was extracted and the most recent year on which the occurrence record was recorded in the grid cell | WOODIV_v2_Occurrence_data.csv  [UTF-8 encoded] |
|  | Occurrence_sources | This table matches the occurrence source code to the source’s information from where occurrences were extracted from | WOODIV_v2_Occurrence_sources.csv  [UTF-8 encoded] |
|  | Country | This table indicates the occurrence status of species at the country level | WOODIV_v2_Country.csv  [UTF-8 encoded] |
| **TRAITS** | Trait_data | This table includes the individual trait values for the 17 traits extracted from trait databases, with source’s code, together with primary record’s information when available, including coordinates | WOODIV_v2_Trait_data.csv  [UTF-8 encoded] |
|  | Trait_sources | This table matches the trait source code to the source from where trait values were extracted | WOODIV_v2_Trait_sources.csv  [UTF-8 encoded] |
| **PHYLOGENY** | Phylogeny - 210 taxa (spagg) | This file corresponds to the time-calibrated phylogeny of the 210 taxa aggregated at species level (spagg); in Newick format | WOODIV_v2_Phylogeny_spagg.tree |
|  | Phylogeny - 206 taxa (gragg) | This file corresponds to the time-calibrated phylogeny of the 206 taxa aggregated at group level (gragg); in newick format | WOODIV_v2_Phylogeny_gragg.tree |
| **SPATIAL** | 10 × 10 km grid | This folder includes the extracted part of the INPIRE LAEA grid (10 × 10 km) that covers the study area as a polygon shapefile | WOODIV_v2_Grid_epsg3035/ |
|  | Study area shape | This folder includes the study area as a polygon shapefile | WOODIV_v2_Shape_epsg3035/ |

***Supplementary Table 7. Description of the columns within the files of the WOODIV v2 database.***

| **Data file name** | **Column name** | **Column description** |
| --- | --- | --- |
| Species_code | spcode | Taxon indexation code |
|  | taxo_level | Taxonomic level of the taxon in the Médail et al. (2019) checklist, either species or subspecies |
|  | full_scientific_name | Taxon full scientific name |
|  | spagg | Indexation code of the taxon when considering it aggregated at species level (spagg, i.e. not considering sub-species) |
|  | gragg | Indexation code of the taxon when considering it aggregated at group level (gragg, i.e. not considering sub-species and grouping closely related species) |
| Nomenclature | spcode | Taxon indexation code |
|  | class | Taxonomic rank at the Class level |
|  | subclass | Taxonomic rank at the Subclass level |
|  | order | Taxonomic rank at the Order level |
|  | family | Taxonomic rank at the Family level |
|  | genus | Taxonomic rank at the Genus level |
|  | species | Taxonomic rank at the Species level |
|  | authority_species | Authority for the Species level |
|  | subspecies | Taxonomic rank at the Subspecies level, if any |
|  | authority_subspecies | Authority for the Subpecies level, if any |
|  | EUROMED_taxon_name | Synonymy in the Euro+Med PlantBase |
|  | Browicz_taxon_name | Synonymy in the Browicz database |
|  | Kew_taxon_name | Synonymy in the Kew database |
| Endemism | spcode | Taxon indexation code |
|  | endemism | Is the species endemic: 0/ no, 1/ yes, as defined in Médail et al. (2019) |
| Occurrence_data | spcode | Taxon indexation code |
|  | idgrid | Cell identity in the LAEA grid |
|  | occ_source | Code of the source from where the occurrence was retrieved |
|  | last_year | The most recent year on which the occurrence record was recorded in the LAEA grid cell if available (NA otherwise) |
| Occurrence_sources | occ_source | Code of the occurrence source |
|  | occ_full_source | Full information about the source from which the value was extracted, citation and acknowledge to the data provider |
| Country | spcode | Taxon indexation code |
|  | country | Countries and islands considered in the WOODIV v2 database: Albania, Balearic, Corsica, Crete, Croatia, Cyprus, France, Greece, Italy, Southern Macedonia, Malta, Montenegro, Portugal, Sardinia, Sicily, Slovenia, Spain |
|  | status | Status of the species at the country level: I/ introduced, N/ native, N?/ putative native |
| Trait_data | spcode | Taxon indexation code |
|  | trait | Traits name, as defined in Table 2 |
|  | value | The value reported for the trait; unit or category as defined in Table 2 |
|  | trait_source | Code of the source from where the value was extracted |
|  | record_id_in_source | ID of the record in the source database when extracted from a database (NA otherwise) |
|  | ref_in_source | Reference linked to the record in the source when extracted from a database (NA otherwise) |
|  | method | Method used to measure the value of the trait if available (NA otherwise) |
|  | X_EPSG3035 | X coordinate of the location of the measured individual in SCR EPSG 3035 when available (NA otherwise) |
|  | Y_EPSG3035 | Y coordinate of the location of the measured individual in SCR EPSG 3035 when available (NA otherwise) |
| Trait_sources | trait_source | Short name of the source from which the value was extracted |
|  | trait_full_source | Full information about the source from which the value was extracted, citation and acknowledgement to the data provider |
| Grid_epsg3035 | idgrid | Identity of the cell polygon in the LAEA grid |
|  | Xcentroid | X coordinate of the centroid of the cell polygon in SCR EPSG 3035 |
|  | Ycentroid | Y coordinate of the centroid of the cell polygon in SCR EPSG 3035 |
| Shape_epsg3035 | country | Name of the country delimited by the polygon |

**Supplementary Information - R code to reproduce key metrics and figures.**

################################################################################

#

# This script allows to describe the WOODIV v2 data base, i.e., generating key .

# metrics presented in the data paper and reproducing figures ..................

#

# Manuel CARTEREAU - manuel.cartereau@imbe.fr ..................................

# 11/02/2025 ...................................................................

#

################################################################################

##### ----------- 1. Load files ------------------------------------------------

# WOODIV v2 spatial grid .......................................................

grid <- here::here("WOODIV_DB_release_v2", "SPATIAL", "WOODIV_v2_Grid_epsg3035",

"WOODIV_v2_Grid_epsg3035.shp") |>

sf::st_read()

# WOODIV v2 occurrence data ....................................................

occ_v2 <- here::here("WOODIV_DB_release_v2", "OCCURRENCE",

"WOODIV_v2_Occurrence_data.csv") |>

data.table::fread()

# WOODIV v1 occurrence data ....................................................

occ_v1 <- here::here("WOODIV_DB_release_v1", "OCCURRENCE",

"WOODIV_Occurrence_data.csv") |>

data.table::fread()

# WOODIV v1 traits data ........................................................

trait_v1 <- here::here("WOODIV_DB_release_v1", "TRAITS",

"WOODIV_Trait_data.csv") |>

data.table::fread()

# WOODIV v2 traits data ........................................................

trait_v2 <- here::here("WOODIV_DB_release_v2", "TRAITS",

"WOODIV_v2_Trait_data.csv") |>

data.table::fread()

# WOODIV v2 phylogeny aggregated at the 'gragg' level ..........................

phylo <- here::here("WOODIV_DB_release_v2", "PHYLOGENY",

"WOODIV_v2_Phylogeny_gragg.tree") |>

ape::read.tree()

# WOODIV v2 aggregation information ............................................

agg <- here::here("WOODIV_DB_release_v2", "SPECIES",

"WOODIV_v2_Species_code.csv") |>

data.table::fread()

# WOODIV v2 taxonomic information ..............................................

taxo <- here::here("WOODIV_DB_release_v2", "SPECIES",

"WOODIV_v2_Nomenclature.csv") |>

data.table::fread()

# Land mask - not in WOODIV ....................................................

# Comes from https://www.naturalearthdata.com/ .................................

land <- here::here("data", "data_raw", "ne_10m_land", "ne_10m_land.shp") |>

sf::st_read()

##### ----------- 2. Occurrence data -------------------------------------------

### Rough description of occurrence data .......................................

# How many records in v2? ......................................................

nrow(occ_v2)

# How many aggregated records (at the 'gragg' level)? ..........................

occ_v1 |>

dplyr::left_join(agg) |>

dplyr::distinct(Idgrid, source_code, gragg) |>

nrow() # in v1

occ_v2 |>

dplyr::left_join(agg) |>

dplyr::distinct(idgrid, occ_source, gragg) |>

nrow() # in v2

# How many aggregated occurrences (at the 'gragg' level)? ......................

occ_v1 |>

dplyr::left_join(agg) |>

dplyr::distinct(Idgrid, gragg) |>

nrow() # in v1

occ_v2 |>

dplyr::left_join(agg) |>

dplyr::distinct(idgrid, gragg) |>

nrow() # in v2

# How many grid cells? .........................................................

nlevels(as.factor(occ_v1$Idgrid)) # in v1

nlevels(as.factor(occ_v2$idgrid)) # in v2

### Table of occurrence updates ................................................

# Aggregated occurrence data at the 'gragg' level ..............................

online_tab1 <- occ_v2 |>

dplyr::left_join(agg) |>

dplyr::distinct(gragg, occ_source) |>

dplyr::group_by(occ_source) |>

dplyr::summarise(n_gragg = dplyr::n()) |>

dplyr::ungroup() |>

dplyr::left_join(occ_v2 |>

dplyr::left_join(agg) |>

dplyr::distinct(gragg, idgrid, occ_source) |>

dplyr::group_by(occ_source) |>

dplyr::summarise(n_rec = dplyr::n()) |>

dplyr::ungroup()) |>

dplyr::left_join(occ_v2 |>

dplyr::left_join(agg) |>

dplyr::distinct(idgrid, occ_source) |>

dplyr::group_by(occ_source) |>

dplyr::summarise(n_cell = dplyr::n()))

# Export .......................................................................

readr::write_csv(online_tab1,

here::here("outputs", "online_tab1.csv"))

### Plot maps for richness of taxa aggregated at the 'gragg' level and data ....

### increase relative to v1 ....................................................

# Prepare data to plot .........................................................

data_grid <- grid |>

dplyr::left_join(occ_v1 |>

dplyr::rename(idgrid = Idgrid) |>

dplyr::left_join(agg) |>

dplyr::distinct(idgrid, gragg) |>

dplyr::group_by(idgrid) |>

dplyr::summarise(Richness_v1 = dplyr::n())) |>

dplyr::left_join(occ_v2 |>

dplyr::left_join(agg) |>

dplyr::distinct(idgrid, gragg) |>

dplyr::group_by(idgrid) |>

dplyr::summarise(Richness_v2 = dplyr::n())) |>

dplyr::mutate(Increase = ((Richness_v2 - Richness_v1) / Richness_v1)*100)

# How many newly sampled grid cell in v2 compared to v1? .......................

data_grid |>

sf::st_drop_geometry() |>

dplyr::filter(is.na(Increase) & Richness_v2 > 0) |>

nrow()

# Median increase per grid cell ................................................

data_grid |>

dplyr::filter(!is.na(Increase)) |>

dplyr::pull(Increase) |>

median()

# Prepare maps' land background ................................................

bg <- land |>

sf::st_transform(crs = sf::st_crs(grid)) |>

sf::st_crop(sf::st_bbox(grid) |>

sf::st_as_sfc() |>

sf::st_buffer(dist = 100000)) |>

dplyr::select(-c("featurecla", "scalerank", "min_zoom"))

# Plot richness map ............................................................

fig1a <- data_grid |>

dplyr::select(Richness_v2) |>

na.omit() |>

ggplot2::ggplot() +

ggplot2::geom_sf(data = bg, fill = "white") +

ggplot2::geom_sf(ggplot2::aes(fill = Richness_v2), color = "NA") +

ggplot2::coord_sf(datum = sf::st_crs(grid),

xlim = c(sf::st_bbox(bg)$xmin, sf::st_bbox(bg)$xmax),

ylim = c(sf::st_bbox(bg)$ymin, sf::st_bbox(bg)$ymax),

expand = F) +

ggplot2::scale_fill_gradientn(colors = hcl.colors(30, "Geyser")) +

ggplot2::labs(fill = "Richness") +

ggplot2::theme(legend.position = "right") +

ggplot2::theme(axis.text.x = ggplot2::element_blank(),

axis.ticks.x = ggplot2::element_blank(),

axis.text.y = ggplot2::element_blank(),

axis.ticks.y = ggplot2::element_blank()) +

ggplot2::theme(panel.grid.major = ggplot2::element_blank(),

panel.grid.minor = ggplot2::element_blank()) +

ggplot2::theme(panel.border = ggplot2::element_rect(fill = "transparent",

color = "black",

size = 1),

plot.margin = ggplot2::unit(c(0, 0, 0, 0), "pt"))

# Plot increase map ............................................................

fig1b <- ggplot2::ggplot() +

ggplot2::geom_sf(data = bg, fill = "white") +

ggplot2::geom_sf(data = data_grid |>

dplyr::select(idgrid, Increase) |>

dplyr::filter(Increase > 0),

ggplot2::aes(fill = log(Increase)), color = "NA") +

viridis::scale_fill_viridis(direction = -1) +

ggplot2::labs(fill = "Increase") +

ggnewscale::new_scale_fill() +

ggplot2::geom_sf(data = data_grid |>

dplyr::filter(is.na(Increase) & Richness_v2 > 0) |>

dplyr::mutate(Value = "add"),

ggplot2::aes(fill = Value, colour = "NA")) +

ggplot2::scale_fill_manual(values = "red", breaks = "add") +

ggplot2::coord_sf(datum = sf::st_crs(grid),

xlim = c(sf::st_bbox(bg)$xmin, sf::st_bbox(bg)$xmax),

ylim = c(sf::st_bbox(bg)$ymin, sf::st_bbox(bg)$ymax),

expand = F) +

ggplot2::theme(axis.text.x = ggplot2::element_blank(),

axis.ticks.x = ggplot2::element_blank(),

axis.text.y = ggplot2::element_blank(),

axis.ticks.y = ggplot2::element_blank()) +

ggplot2::theme(panel.grid.major = ggplot2::element_blank(),

panel.grid.minor = ggplot2::element_blank()) +

ggplot2::theme(panel.border = ggplot2::element_rect(fill = "transparent",

color = "black",

size = 1),

plot.margin = ggplot2::unit(c(0, 0, 0, 0), "pt")) +

ggplot2::theme(legend.position = "right") +

ggplot2::guides(color = "none",

fill = "none")

# Gather the two maps and export ...............................................

fig1 <- egg::ggarrange(fig1a, fig1b, ncol = 1, nrow = 2)

ggplot2::ggsave(fig1,

filename = here::here("figures", "fig1.png"),

device = "png", width = 17, height = 15, units = "cm",

dpi = 500)

##### ----------- 3. Traits data -----------------------------------------------

# Traits availability at the 'gragg' level in WOODIV v1 and v2 .................

traits_gragg_v1_v2 <- trait_v1 |>

dplyr::filter(Traits != "Height") |>

dplyr::left_join(agg) |>

dplyr::distinct(Traits, gragg) |>

dplyr::mutate(in_v1 = 1) |>

dplyr::rename(Trait = Traits) |>

dplyr::full_join(trait_v2 |>

dplyr::left_join(agg) |>

dplyr::distinct(trait, gragg) |>

dplyr::mutate(in_v2 = 1) |>

dplyr::rename(Trait = trait))

# How many gragg x traits combinations in v1? ..................................

traits_gragg_v1_v2 |>

na.omit() |>

nrow()

# How many gragg x traits combinations in v2? ..................................

traits_gragg_v1_v2 |>

nrow()

# How many gragg with at least one trait record in v1? .........................

graggv1 <- traits_gragg_v1_v2 |>

na.omit() |>

dplyr::distinct(gragg) |>

nrow()

# How many gragg with at least one trait record in v2? .........................

graggv2 <- traits_gragg_v1_v2 |>

dplyr::distinct(gragg) |>

nrow()

# Mean traits completeness in v1? ..............................................

traits_gragg_v1_v2 |>

dplyr::mutate(in_v1 = dplyr::case_when(is.na(in_v1) ~ 0,

.default = in_v1)) |>

dplyr::group_by(gragg) |>

dplyr::summarise(n = sum(in_v1)) |>

dplyr::pull(n) |>

median() / 3 * 100

# Mean traits completeness in v2? ..............................................

traits_gragg_v1_v2 |>

dplyr::group_by(gragg) |>

dplyr::summarise(n = sum(in_v2)) |>

dplyr::pull(n) |>

median() / 17 * 100

# How many gragg with at least one trait record in v2? .........................

traits_gragg_v1_v2 |>

dplyr::distinct(gragg) |>

nrow()

# Traits completeness at the 'gragg' level in WOODIV v1 and v2 .................

traits_gragg_v1_v2_comp <- traits_gragg_v1_v2 |>

dplyr::group_by(Trait) |>

dplyr::summarise(Ngragg_v2 = dplyr::n(),

Perc_v2 = ((dplyr::n()) / 206)*100) |>

dplyr::left_join(traits_gragg_v1_v2 |>

dplyr::filter(!is.na(in_v1)) |>

dplyr::group_by(Trait) |>

dplyr::summarise(Ngragg_v1 = dplyr::n(),

Perc_v1 = ((dplyr::n()) / 206)*100)) |>

tidyr::replace_na(list(Perc_v1 = 0, Ngragg_v1 = 0)) |>

dplyr::mutate(Perc_inc = Perc_v2 - Perc_v1)

# Species completeness at the 'gragg' level in terms of percentage of traits in

# v2 ...........................................................................

gragg_traits_v2_comp <- trait_v2 |>

dplyr::left_join(agg) |>

dplyr::distinct(gragg, trait) |>

dplyr::group_by(gragg) |>

dplyr::summarize(n_trait = dplyr::n()) |>

dplyr::mutate(trait_comp = round(n_trait / 17, digits = 2)*100) |>

dplyr::arrange(dplyr::desc(trait_comp))

### Plot traits completeness ...................................................

# Prepare data .................................................................

dat <- traits_gragg_v1_v2_comp |>

dplyr::select(-Perc_v2) |>

tidyr::pivot_longer(!Trait, names_to = "Version", values_to = "Perc") |>

dplyr::filter(Version == "Perc_inc" | Version == "Perc_v1") |>

dplyr::mutate(Version = dplyr::case_match(Version, "Perc_v1" ~ "v1",

"Perc_inc" ~ "v2")) |>

dplyr::mutate(Version = relevel(as.factor(Version), "v2")) |>

dplyr::mutate(Trait = factor(Trait, levels = c("BloomBreadth", "BloomPosition",

"BloomEnd", "BloomStart", "LeafPheno",

"SeedMass", "SexSys",

"Pollination", "DispDist",

"DispMode", "StemSpecDens",

"HeightMax", "SLA", "LeafArea",

"LeafOutline", "LeafMargin",

"LeafShape")))

# Number of aggregated taxa at the 'gragg' level having information for a given

# trait without considering main traits repositories, i.e., TRY, BIEN and BROT .

# in WOODIV v2 .................................................................

without_main_repo_v2 <- trait_v2 |>

dplyr::filter(trait_source != "TRY" & trait_source != "BIEN" &

trait_source != "BROT") |>

dplyr::left_join(agg) |>

dplyr::distinct(gragg, trait) |>

dplyr::rename(Trait = trait) |>

dplyr::group_by(Trait) |>

dplyr::summarise(Ngragg_without = dplyr::n()) |>

dplyr::left_join(traits_gragg_v1_v2_comp |>

dplyr::select(Trait, Ngragg_v2, Perc_v2)) |>

dplyr::rename(Ngragg_with = Ngragg_v2) |>

dplyr::mutate(Perc = round((Ngragg_without / Ngragg_with)*100, digits = 0))

sum(without_main_repo_v2$Ngragg_without)

# Plot .........................................................................

fig2a <- dat |>

ggplot2::ggplot() +

ggplot2::geom_col(ggplot2::aes(x = Trait, y = Perc, fill = Version),

width = .9) +

ggplot2::geom_text(data = without_main_repo_v2 |>

dplyr::select(Trait, Ngragg_with, Perc_v2, Perc),

ggplot2::aes(x = Trait, y = Perc_v2,

label = paste0(Ngragg_with, " (excl.db. ", Perc,

"%)")),

hjust = 1.2, color = "white") +

ggplot2::scale_fill_manual(values = c("royalblue4", "gray48")) +

ggplot2::coord_flip() +

ggplot2::theme_minimal() +

ggplot2::ylab("Completeness (% of aggregated taxa)") +

ggplot2::xlab(NULL) +

ggplot2::theme(axis.text.x = ggplot2::element_text(size = 12),

axis.text.y = ggplot2::element_text(face = "bold", size = 14),

legend.position = "none")

### Plot traits completeness on phylogeny at the 'gragg' level in terms of % of

### traits with at least one record ............................................

### Inspect taxonomy to find main clades .......................................

taxo2 <- taxo |>

dplyr::left_join(agg) |>

dplyr::distinct(gragg, .keep_all = T) |>

dplyr::select(gragg, order, family, genus)

# Orders .......................................................................

ord <- taxo2 |>

dplyr::group_by(order) |>

dplyr::summarise(nagg = dplyr::n()) |>

dplyr::arrange(desc(nagg))

# Families .....................................................................

fam <- taxo2 |>

dplyr::group_by(family) |>

dplyr::summarise(nagg = dplyr::n()) |>

dplyr::arrange(desc(nagg))

# Genera .......................................................................

gen <- taxo2 |>

dplyr::group_by(genus) |>

dplyr::summarise(nagg = dplyr::n()) |>

dplyr::arrange(desc(nagg))

# Find MRCA of main families ...................................................

# Rosaceae .....................................................................

ros <- ape::getMRCA(phylo, tip = dplyr::filter(taxo2, family == "Rosaceae")[["gragg"]])

# Fagaceae .....................................................................

fag <- ape::getMRCA(phylo, tip = dplyr::filter(taxo2, family == "Fagaceae")[["gragg"]])

# Salicaceae ...................................................................

sal <- ape::getMRCA(phylo, tip = dplyr::filter(taxo2, family == "Salicaceae")[["gragg"]])

# Tamaricaceae .................................................................

tam <- ape::getMRCA(phylo, tip = dplyr::filter(taxo2, family == "Tamaricaceae")[["gragg"]])

# Pinaceae .....................................................................

pin <- ape::getMRCA(phylo, tip = dplyr::filter(taxo2, family == "Pinaceae")[["gragg"]])

# Betulaceae ...................................................................

bet <- ape::getMRCA(phylo, tip = dplyr::filter(taxo2, family == "Betulaceae")[["gragg"]])

# Sapindaceae ..................................................................

sap <- ape::getMRCA(phylo, tip = dplyr::filter(taxo2, family == "Sapindaceae")[["gragg"]])

# Cupressaceae .................................................................

cup <- ape::getMRCA(phylo, tip = dplyr::filter(taxo2, family == "Cupressaceae")[["gragg"]])

# Oleaceae .....................................................................

ole <- ape::getMRCA(phylo, tip = dplyr::filter(taxo2, family == "Oleaceae")[["gragg"]])

# Ulmaceae .....................................................................

ulm <- ape::getMRCA(phylo, tip = dplyr::filter(taxo2, family == "Ulmaceae")[["gragg"]])

# Fabaceae .....................................................................

fab <- ape::getMRCA(phylo, tip = dplyr::filter(taxo2, family == "Fabaceae")[["gragg"]])

# Anacardiaceae ................................................................

ana <- ape::getMRCA(phylo, tip = dplyr::filter(taxo2, family == "Anacardiaceae")[["gragg"]])

# Design the tree ..............................................................

plotphylo <- ggtree::ggtree(phylo, size = 0.2, layout = "fan", open.angle = 1)

# Highlight families with >= 5 aggregates ......................................

plotphylo2 <- plotphylo +

ggtree::geom_hilight(node = ros, fill = "black", alpha = 0.2) +

ggtree::geom_hilight(node = fag, fill = "black", alpha = 0.2) +

ggtree::geom_hilight(node = sal, fill = "grey", alpha = 0.2) +

ggtree::geom_hilight(node = tam, fill = "grey", alpha = 0.2) +

ggtree::geom_hilight(node = pin, fill = "black", alpha = 0.2) +

ggtree::geom_hilight(node = bet, fill = "grey", alpha = 0.2) +

ggtree::geom_hilight(node = sap, fill = "black", alpha = 0.2) +

ggtree::geom_hilight(node = cup, fill = "grey", alpha = 0.2) +

ggtree::geom_hilight(node = ole, fill = "black", alpha = 0.2) +

ggtree::geom_hilight(node = ulm, fill = "grey", alpha = 0.2) +

ggtree::geom_hilight(node = fab, fill = "black", alpha = 0.2) +

ggtree::geom_hilight(node = ana, fill = "grey", alpha = 0.2)

# Add traits completeness ......................................................

library(ggplot2)

fig2b <- plotphylo2 +

ggtreeExtra::geom_fruit(data = gragg_traits_v2_comp |>

tidyr::pivot_longer(!gragg,

names_to = "Metric",

values_to = "Value") |>

dplyr::filter(Metric == "trait_comp"),

geom = geom_tile,

mapping = ggplot2::aes(y = gragg,

x = Metric,

fill = Value),

offset = 0.015,

pwidth = 15) +

ggplot2::scale_fill_continuous(type = "viridis", direction = -1) +

ggplot2::labs(fill = NULL) +

ggplot2::theme(legend.position = c(0.50, 0.4),

legend.direction = "horizontal")

### Export figures .............................................................

ggplot2::ggsave(fig2b,

filename = here::here("figures", "fig2b.png"),

device = "png", width = 16, height = 16, units = "cm",

dpi = 500)

ggplot2::ggsave(fig2a,

filename = here::here("figures", "fig2a.png"),

device = "png", width = 10, height = 16, units = "cm",

dpi = 500)

R version 4.3.2 (2023-10-31 ucrt)

Platform: x86_64-w64-mingw32/x64 (64-bit)

Running under: Windows 11 x64 (build 26100)

Matrix products: default

locale:

[1] LC_COLLATE=French_France.utf8 LC_CTYPE=French_France.utf8 LC_MONETARY=French_France.utf8 LC_NUMERIC=C LC_TIME=French_France.utf8

time zone: Europe/Paris

tzcode source: internal

attached base packages:

[1] stats graphics grDevices utils datasets methods base

other attached packages:

[1] ggplot2_3.5.1

loaded via a namespace (and not attached):

[1] gtable_0.3.6 lattice_0.21-9 tzdb_0.4.0 vctrs_0.6.4 tools_4.3.2 generics_0.1.3 yulab.utils_0.1.4 parallel_4.3.2 tibble_3.2.1

[10] proxy_0.4-27 pkgconfig_2.0.3 KernSmooth_2.23-22 data.table_1.14.8 ggnewscale_0.4.9 ggplotify_0.1.2 lifecycle_1.0.4 farver_2.1.2 compiler_4.3.2

[19] textshaping_0.3.7 treeio_1.26.0 egg_0.4.5 munsell_0.5.1 ggtreeExtra_1.12.0 ggtree_3.10.0 ggfun_0.1.4 class_7.3-22 lazyeval_0.2.2

[28] crayon_1.5.3 pillar_1.10.1 tidyr_1.3.0 classInt_0.4-10 cachem_1.0.8 viridis_0.6.5 nlme_3.1-163 tidyselect_1.2.1 aplot_0.2.2

[37] digest_0.6.33 sf_1.0-17 dplyr_1.1.4 purrr_1.0.2 labeling_0.4.3 rprojroot_2.0.4 fastmap_1.1.1 grid_4.3.2 here_1.0.1

[46] colorspace_2.1-0 cli_3.6.1 magrittr_2.0.3 patchwork_1.2.0 e1071_1.7-14 ape_5.7-1 withr_3.0.2 readr_2.1.4 scales_1.3.0

[55] bit64_4.0.5 bit_4.0.5 gridExtra_2.3 ragg_1.2.7 hms_1.1.3 memoise_2.0.1 viridisLite_0.4.2 gridGraphics_0.5-1 rlang_1.1.2

[64] Rcpp_1.0.11 glue_1.6.2 tidytree_0.4.6 DBI_1.2.2 rstudioapi_0.17.1 vroom_1.6.5 jsonlite_1.8.8 R6_2.5.1 systemfonts_1.0.5

[73] fs_1.6.5 units_0.8-5
